# Kinetic Analysis of the Interaction of Nicking Endonuclease BspD6I with DNA

**DOI:** 10.3390/biom11101420

**Published:** 2021-09-28

**Authors:** Liudmila A. Abrosimova, Nikita A. Kuznetsov, Natalia A. Astafurova, Anastasiia R. Samsonova, Andrey S. Karpov, Tatiana A. Perevyazova, Tatiana S. Oretskaya, Olga S. Fedorova, Elena A. Kubareva

**Affiliations:** 1 Department of Chemistry, Lomonosov Moscow State University, Leninskie Gory 1, 119991 Moscow, Russia; nataliaandreeva93@hotmail.com (N.A.A.); etudiantmsu@yandex.ru (A.S.K.); 2 Institute of Chemical Biology and Fundamental Medicine, Siberian Branch of Russian Academy of Sciences, Lavrentiev Avenue 8, 630090 Novosibirsk, Russia; fedorova@niboch.nsc.ru; 3 SAS Synhelix, Genopole Campus 3, 4 Rue Pierre Fontaine, 91058 Evry-Courcouronnes, France; one.two.three908@gmail.com; 4 Institute of Theoretical and Experimental Biophysics, Russian Academy of Sciences, Institutskaya Str. 3, 142290 Puschino, Russia; anatolia19@mail.ru; 5 Belozersky Institute of Physico-Chemical Biology, Lomonosov Moscow State University, Leninskie Gory 1, 119991 Moscow, Russia; oretskaya@belozersky.msu.ru (T.S.O.); kubareva@belozersky.msu.ru (E.A.K.)

**Keywords:** nicking endonuclease, pre–steady-state kinetics, DNA-protein interaction, kinetic mechanism

## Abstract

Nicking endonucleases (NEs) are enzymes that incise only one strand of the duplex to produce a DNA molecule that is ‘nicked’ rather than cleaved in two. Since these precision tools are used in genetic engineering and genome editing, information about their mechanism of action at all stages of DNA recognition and phosphodiester bond hydrolysis is essential. For the first time, fast kinetics of the Nt.BspD6I interaction with DNA were studied by the stopped-flow technique, and changes of optical characteristics were registered for the enzyme or DNA molecules. The role of divalent metal cations was estimated at all steps of Nt.BspD6I–DNA complex formation. It was demonstrated that divalent metal ions are not required for the formation of a non-specific complex of the protein with DNA. Nt.BspD6I bound five-fold more efficiently to its recognition site in DNA than to a random DNA. DNA bending was confirmed during the specific binding of Nt.BspD6I to a substrate. The optimal size of Nt.BspD6I’s binding site in DNA as determined in this work should be taken into account in methods of detection of nucleic acid sequences and/or even various base modifications by means of NEs.

## 1. Introduction

Nicking endonucleases (NEs) are enzymes that recognise specific sequences in double-stranded DNA (dsDNA), but unlike restriction endonucleases (REs, Rs), NEs cleave only one DNA strand at a certain position relative to the recognition site. The first NE was discovered in 1996 in the thermophilic microorganism *Bacillus stearothermophilus* SE-589 [1]. Currently, according to the REBASE (http://rebase.neb.com/cgi-bin/azlist?nick, accessed on 21 September 2021), more than 14,000 NEs are known (including putative enzymes). Only 15 of them are commercially available. Most of the characterised NEs are from bacteria of the genus *Bacillus*. 

The NEs that catalyse the cleavage of the top or bottom DNA strand in the duplex (5′→3′/3′→5′) are denoted as Nt (‘nicking top’) and Nb (‘nicking bottom’), respectively. The discovery of NEs has allowed researchers to improve or simplify several methods of molecular biology [2] such as isothermal DNA amplification [2,3], site-directed mutagenesis [2], DNA labelling, and the introduction of modifications into DNA [2,3]. NEs are used for the construction of highly specific chimeric proteins and the engineering of genetic constructs for the exploration of biological processes [3]; NEs’ ability to cleave only one DNA strand has become the basis for various techniques of detection of small-molecule compounds, ions, biopolymers, and cells [3].

The aim of the present work was to investigate the interaction of NE BspD6I (Nt.BspD6I) with DNA under pre–steady-state conditions, namely, to determine the order of conformational changes in the enzyme and in the DNA substrate during the reaction. This work is relevant because despite the research on NEs for more than 20 years and their broad application in biotechnology [3], the precise mechanism of their interaction with DNA is still not fully understood. This state of affairs can slow the development of new methods based on NEs and the creation of chimeric proteins with altered specificity. In bacterial repair and recombination systems, there are enzymes functionally similar to NEs—for example, MutL proteins from the mismatch repair system (MMR). Unlike NEs, they are extremely conformationally flexible and labile, and that is why it is difficult to study the intricate mechanisms of their action. Another example of site-specific DNA-cleaving enzymes is apurinic/apyrimidinic (AP) endonucleases, which are some of the key DNA repair enzymes in the base excision repair pathway [4]. Based on pre–steady-state kinetic analysis, it has been suggested that the key factor responsible for the substrate specificity of AP endonucleases is the ability of a damaged nucleotide to become everted from a double helix and inserted into the damaged-nucleotide-binding pocket [5]. It can be hypothesised that the features of the mechanism underlying the introduction of a single-strand break into DNA may be common among all the above-mentioned enzymes. To test this hypothesis in the present study, Nt.BspD6I from thermophilic bacterial strain *Bacillus species D6* was analysed [6]. 

**Figure 1:**
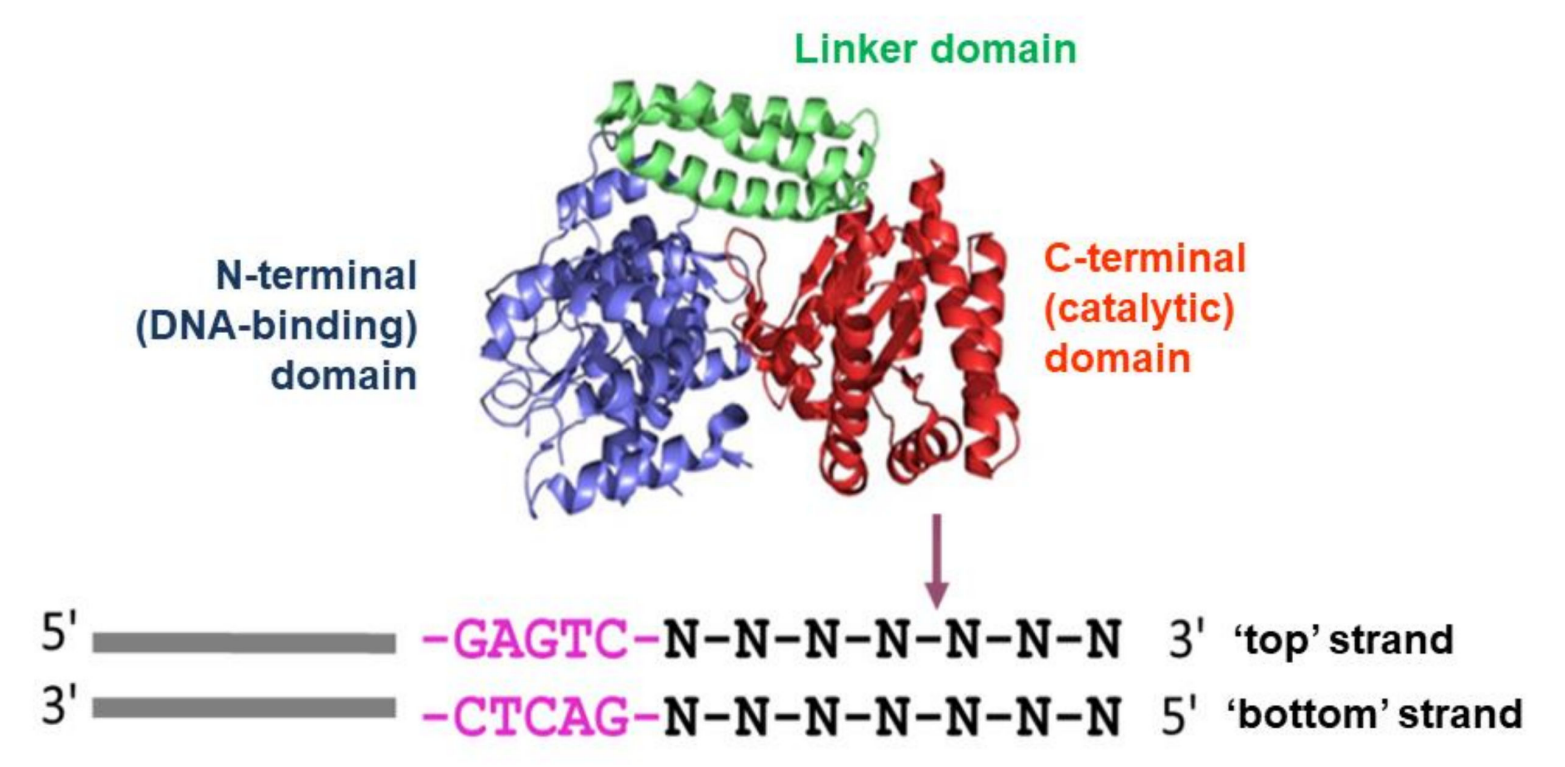
An outline of the domain organisation of Nt.BspD6I (Protein Data Bank ID: 2EWF), the enzyme’s recognition site (highlighted in pink), and the cleavage site in the top DNA strand (arrow).

In dsDNA, Nt.BspD6I recognises the 5′-GAGTC-3′/5′-GACTC-3′ sequence, which is frequent in promoters of phage genes (e.g., T7 bacteriophage). Nt.BspD6I catalyses the hydrolysis of a phosphodiester bond in the top strand after the fourth nucleotide from the recognition site towards the 3′ end (Figure 1) [7].

Nt.BspD6I contains 604 amino acid residues, and its molecular mass is 70.8 kDa. The 3D structure of the protein has been obtained at high resolution (1.8 Å [8]). Nt.BspD6I has a shape of a compact globule (50 × 60 × 80 Å) in which three domains can be distinguished: N-terminal (comprising two subdomains), linker, and C-terminal (Figure 1). Based on the structural similarity of Nt.BspD6I with the well-studied RE FokI, it has been suggested that the Nt.BspD6I N-terminal domain is DNA-binding, whereas the C-terminal domain is catalytic. The linker domain of Nt.BspD6I is probably important for fixing the exact distance between the DNA-binding domain and catalytic domain [8].

To date, the attempts to crystallise the Nt.BspD6I complex with DNA have not been successful; however, some aspects of the complex structure have been studied. The authors of ref. [9], using atomic force microscopy, confirmed the location of the enzyme at a specific site after its binding to DNA duplexes with a length of 920 bp adsorbed on a mica layer in the presence of Mg^2+^ ions. In our group, using the circular permutation method based on differences in the mobility of DNA–protein complexes during polyacrylamide gel electrophoresis (PAGE), Nt.BspD6I has been shown to induce DNA bending by an angle of 66° ± 4° during complex formation and to be able to form a complex with the product of nicking [10]. Some contacts in the Nt.BspD6I complex with DNA have been predicted, and an approach to the regulation of the Nt.BspD6I activity by means of synthetic DNA duplexes has been proposed [11]. On the other hand, the molecular–kinetic mechanism of Nt.BspD6I action, the knowledge about which is necessary for complete characterisation of nucleic acid–protein interactions, has not been researched yet. Information about the mechanism of functioning of other NEs in the literature is also lacking. The only paper was published in 2019, where a model of substrate interaction of Nt.BstNBI (isoschizomer of Nt.BspD6I) was suggested using locked-nucleic-acid substrate analogues and the rolling circle amplification technique [12]. We should mention that the data presented in ref. [12] are in line with our earlier results on Nt.BspD6I [10,11]. The structural organisation of Nt.BspD6I [8] and results from ref. [12] have further confirmed the existence of separate recognition and cleavage domains in Nt.BstNBI from a biochemical point of view and indicate that the catalytic domain of this NE has great potential for the construction of chimeric endonucleases containing recognition domains with other sequence specificity.

Even though structural methods make a huge contribution to the understanding of the nature of enzymatic catalysis, they are largely limited to describing only a few fixed conformational states of an enzyme and its substrate during an enzymatic reaction. Meanwhile, evidence of conformational transitions in enzymes and substrates, together with structural information, is crucial for the creation of adequate models of enzymatic catalysis. One of the most substantial contributions to the elucidation of these issues is made by approaches involving pre–steady-state kinetics of conformational transitions of interacting molecules during an enzymatic process.

Therefore, in this work, for the first time, we studied the fast kinetics of the Nt.BspD6I interaction with DNA by the stopped-flow technique, and the changes of optical characteristics were registered for the enzyme and various DNA molecules. A molecular mechanism of the Nt.BspD6I interaction with DNA (complex formation, cleavage of one strand of dsDNA, and a product release) is proposed for the first time. We showed that the minimal kinetic scheme of the Nt.BspD6I interaction with DNA in the presence of Mg^2+^ includes several stages: non-specific DNA binding, a search for the recognition site, the formation of a specific enzyme–substrate complex accompanied by DNA bending, catalysis, and dissociation of the enzyme from the product. We also investigated the influence of the length of the DNA sequences flanking the recognition site and the impact of two divalent cations acting as cofactors on the kinetics of the Nt.BspD6I–DNA interaction. After comparing our data on Nt.BspD6I with the literature data concerning the mechanism of action of some type II REs, we can conclude that their activities have common features.

## 2. Materials and Methods

### 2.1. Proteins and DNA Fragments

Nt.BspD6I was expressed and purified as described previously [10] and was stored in buffer A (20 mM HEPES-NaOH, 100 mM NaCl, 0.1 mM EDTA, 50% [*w*/*v*] of glycerol, pH 7.5). Bovine serum albumin (BSA) was purchased from Thermo Fisher Scientific (Waltham, MA, USA).

All oligonucleotides were synthesised by Syntol (Moscow, Russia), Evrogen (Moscow, Russia), or the Institute of Chemical Biology and Fundamental Medicine (the Siberian Branch of the Russian Academy of Sciences, Novosibirsk, Russia). Nt.BspD6I and oligonucleotide concentrations were determined spectrophotometrically. 

Solutions of the DNA duplexes used in this work (Table 1) were prepared from samples of complementary oligonucleotides mixed in equimolar amounts by heating them to 90 °C and then slowly cooling to room temperature.

### 2.2. Nicking of DNA Duplex VI-30 by Nt.BspD6I in the Presence of Divalent Metal Ions 

DNA cleavage by the enzyme was performed during 30 min at 37 °C in 10 mM Tris-HCl buffer (pH 7.8) supplemented with 150 mM KCl. The reaction contained 10 nM DNA duplex and 10 nM enzyme, and the concentration of Zn^2+^, Cd^2+^, Ni^2+^, Mn^2+^, Co^2+^, or Mg^2+^ varied from 5 to 15 mM. Buffer B (TBE buffer (50 mM Tris-HCl, 50 mM boric acid, 1 mM EDTA, pH 8.3), 50 mM EDTA, and 2% of SDS) was employed to stop the reaction.

After the nicking reaction, the mixtures were separated by 20% PAGE with a plate size of 200 × 200 × 1 mm^3^ (acrylamide:N,N′-bisacrylamide at 19:1) in the presence of 7 M urea at a field force of 30 V/cm in TBE buffer [13]. Before loading onto the gel, the reaction mixtures were heated for 3 min at 95 °C. On a Typhoon FLA 9500 instrument (GE Healthcare, USA), the fluorescence of the zones containing DNA was detected and analysed using the ImageQuant software (GE Healthcare, UK) (https://www.cytivalifesciences.com/en/us/shop/protein-analysis/molecular-imaging-for-proteins/imaging-software/imagequant-tl-8-2-image-analysis-software-p-09518, accessed on 21 September 2021). The extent of DNA nicking (percentage cleavage) was defined as the ratio of the product to the total DNA. Each measurement was performed at least three times; standard error (SE) did not exceed 8–10%. Standard error was calculated in Origin (OriginLab, Northampton, MA, USA, https://www.originlab.com/, accessed on 21 September 2021) according to the formula SE = s/n^0.5^, where SE: standard error, s: standard deviation, and n: the number of independent experiments (at least three).

### 2.3. DNA Duplex **II-19A** Cleavage by Nt.BspD6I for an Analysis of Steady-State Kinetics of the Reaction 

This experiment was carried out for 30 min at 37 °C. To obtain the time-dependences of the substrate nicking extent, 126 μL of a solution containing 40, 50, 65, 85, 200, 300, or 400 nM DNA substrate, 1 mM DTT and 0.1% of BSA in buffer C (10 mM Tris-HCl, 150 mM KCl, 10 mM MgCl_2_, pH 7.8) was mixed with 14 μL of a 70 nM enzyme solution. After rapid stirring of the reaction mixture, 10 μL aliquots were withdrawn from it at 1, 3, 5, 7, 10, 15, 20, 25, and 30 min after the start of the reaction and were placed in prepared tubes containing 3 μL of buffer B to stop the reaction [14]. The reaction mixtures were analysed as described above.

The initial rates of cleavage (*v*_0_) of DNA duplexes by Nt.BspD6I were calculated as the tangent of the slope of the initial linear portion of the kinetic curve; this parameter was determined graphically using the Origin software. Then, in the same software, the dependence of the initial cleavage rate (*v*_0_) on the concentration of the DNA substrate [S] was plotted, which at low substrate concentrations is described by the equation *v*_0_ = (*k_cat_*/*K_M_*)⋅[E]_0_⋅[S] = (*V_max_*/*K_M_*)⋅[S], where *k_cat_* is the catalytic constant of the reaction rate, *K_M_* is the Michaelis constant, [E]_0_ denotes the initial concentration of the enzyme, and *V_max_* means the maximum reaction rate [15]. The tangent of the slope of the straight-line segment of this dependence (V_max_/*K_M_*) was determined graphically, and the ratio of the catalytic-reaction rate constant *k_cat_* to Michaelis constant *K_M_* (*k_cat_*/*K_M_*) was calculated as the ratio of *V_max_*/*K_M_* to enzyme concentration [E]_0_. *K_M_* and *V_max_* were determined in Origin after the fitting of experimental data to the Michaelis–Menten equation; *k_cat_* was calculated from the obtained *k_cat_*/*K_M_* and *K_M_* [16].

To evaluate the effect of a fluorescent label on the reaction kinetics, the cleavage of DNA duplex **II-19A** by Nt.BspD6I was performed as described above, via mixing of solutions containing 200 nM DNA duplex and 40 nM enzyme. In the case of ^32^P-labelled DNA substrates, after electrophoretic separation of the reaction products, a gel autoradiograph was produced, which was analysed by the ImageQuant software. From the obtained kinetic dependences, the half-life of the substrate was calculated.

### 2.4. Pre–Steady-State Kinetics of the Interaction of Nt.BspD6I with a DNA Duplex 

The stopped-flow method was utilised on an SX.18MV spectrometer (Applied Photophysics, UK). Solutions of DNA (0.1–1.5 μM) and the enzyme (0.25–1.50 μM) were placed in syringes and were mixed automatically in a temperature-controlled cuvette. The total volume of the reaction mixture in the cuvette was 100 μL. The ‘dead’ time of the device—that is, the time of mixing the solutions in the syringes—is 1.38 ms. A photomultiplier with an installed light filter served as an integrated intensity detector. Excitation and emission wavelengths of the reporter groups (carboxyfluorescein (FAM) and Black Hole Quencher 1 (BHQ1)) used in the work and the light filters are listed in Table S1 (Supplementary Materials) [17].

The intrinsic fluorescence of Nt.BspD6I was recorded as the fluorescence of Trp residues. To register conformational changes in DNA substrates, the pair of the FAM fluorophore and BHQ1 fluorescence quencher was used. 

The reaction was carried out at 37 °C in three buffers: solution C contained Mg^2 +^, which is necessary for the catalysis of the hydrolysis reaction; solution D (10 mM Tris-HCl, 150 mM KCl, 10 mM CaCl_2_, pH 7.8) contained Ca^2+^, in the presence of which an unproductive enzyme–substrate complex forms; solution E (10 mM Tris-HCl, 150 mM KCl, 0.4 mM EDTA, pH 7.8) did not contain divalent metal ions. The maximum recording time for fluorescence intensity changes was 1000 s. Each kinetic curve was built by averaging at least three independently obtained experimental curves.

### 2.5. Quantitative Analysis of the Results of the Kinetic Experiments Conducted under Pre–Steady-State Conditions 

A series of kinetic curves for the interaction of equimolar Nt.BspD6I and one of DNA duplexes of various lengths (**I-17**, **II-19**, **III-21**, **IV-23**, **V-26,** or **VI-30**) were fitted to the following sum of two exponentials by the method of non-linear regression in the Origin software:

(1) $$y=C+A_{1}e^{-k_{1}x}+A_{2}\left(1-e^{-k_{2}x}\right)$$

The observed rate constants of several stages in the process of the enzyme–substrate complex formation were determined next.

The concentration series of kinetic curves were analysed by means of the Origin and DynaFit software (BioKin, USA) [18] by the method of numerical integration of a system of differential equations describing the kinetics of the process (non-linear regression analysis, Levenberg–Marquardt algorithm [18]), including the optimisation of parameters in the kinetic schemes. The approach is based on the fluorescence intensity variation in the course of the reaction owing to the sequential formation and subsequent transformation of a DNA–enzyme complex. The kinetic parameters (both the rate constants and response factors) were obtained by global non-linear fitting procedures using the DynaFit software (BioKin, Pullman, WA) [18]. The software performs numerical integration of a system of differential equations with subsequent non-linear least-squares regression analysis. The response factors of the intermediates (which are essentially mathematical products of the extinction coefficients and the fluorescence quantum yields) were regarded as fitting parameters during the data processing.

In the data processing, the kinetic information is obtained from temporal behaviour of fluorescence intensity, not from amplitudes of specific signal contributions. The ‘response factors’ for different DNA–enzyme complexes resulting from the fits provide additional information on fluorescence intensity variations in different complexes.

Processing of individual kinetic curves does not unambiguously provide the kinetic parameters; therefore, global fits of sets of kinetic curves obtained at different concentrations of the reactants were used. During the fitting, all relevant rate constants for the forward and reverse reactions as well as specific molar responses for all intermediate complexes were optimised.

**Scheme 1:**
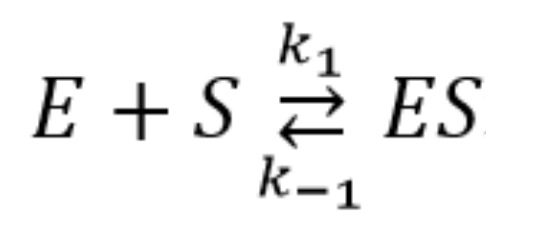
The kinetic schemes of the biochemical reactions 1.

**Scheme 2:**
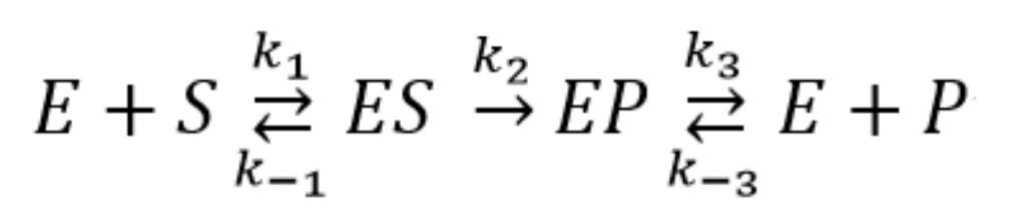
The kinetic schemes of the biochemical reactions 2.

Several mechanisms containing one or more reversible binding steps were evaluated. The procedure of the mechanism determination has been described in detail in previous publications [19,20]. The criterion for validation of the results was the deviation of the calculated theoretical curves from the experimental data. Below are the kinetic schemes employed in this work (E: enzyme (Nt.BspD6I), S: a DNA substrate, ES: an enzyme–substrate complex, EP: a complex of the enzyme with a reaction product, and P: a reaction product) (Scheme 1 and Scheme 2).

*k_cat_* and *K_M_* for Scheme 2 were calculated by means of the following formulas according to the equations obtained using the graph theory:

(2) $$k_{cat}=k_{2}$$

*K_M_* = (*k*_2_*k*_3_ + *k*_−1_*k*_3_ + *k*_2_*k*_−3_)/*k*_1_*k*_3_ (3)

## 3. Results and Discussion

### 3.1. Evaluation of the Methods for Assaying the Kinetics of the Nt.BspD6I Interaction with DNA

It is known that the recognition of DNA substrates by REs and their cleavage reaction occur in millisecond and second ranges [21,22,23]; therefore, the ‘stopped-flow’ approach, which is routine for studying conformational transformations of biomolecules, was chosen to identify the mechanism of Nt.BspD6I action. It allows not only to quickly mix solutions of the enzyme and substrate (‘dead’ time was 1.4 ms in this study) but also to continuously record changes in optical properties of interacting molecules, thus yielding their time dependence.

The optimal temperature for Nt.BspD6I function is 55 °C [24]. On the other hand, the model DNA duplexes tested here are rather short, and at this temperature, they can dissociate into their constituent single-stranded oligos because the melting points of some of them are below 55 °C (for example, 51 °C for **II-19A**). To prevent the melting of DNA duplexes during the reaction, the experiments were carried out at 37 °C. It has been proven earlier [10] that Nt.BspD6I has rather high activity at this temperature.

It is convenient to examine the conformational rearrangements of an enzyme during the reaction by means of the fluorescence of its Trp and/or Tyr residues [25] because the introduction of additional groups into the enzyme molecule complicates the experimental procedure and can change the mechanism and kinetics of enzymatic activity. Nonetheless, the main contribution to protein fluorescence is made by Trp residues [26,27,28,29]. Nt.BspD6I contains seven Trp residues located in various regions of the protein molecule. Some of these residues may be situated near the DNA-binding and catalytic centres [8] (Figure S1). When a protein conformation changes, the environment of Trp residues can also change, which in turn can lead to a variation in the intensity of their fluorescence signals. It is important to understand that structural alterations in the conformation of an enzyme do not necessarily affect Trp residues.

To detect conformational changes in DNA substrates, a fluorophore–fluorescence quencher pair can be utilised [21,30,31,32,33]. A quantitative analysis of the kinetic curves obtained by the stopped-flow method can produce several sets of elementary kinetic constants that describe the experimental data equally well. Therefore, to verify the kinetic schemes obtained by this method (Section 2.4 and Section 2.5), we decided to also determine steady-state reaction parameters: Michaelis constant *K_M_* and catalytic reaction rate constant *k_cat_*.

Enzymatic processing of nucleic acids requires the presence of a divalent metal ion cofactor. To choose optimal conditions for the kinetic experiments at the stages of Nt.BspD6I binding and catalysis, we first needed to investigate the cofactor requirements for this enzyme.

### 3.2. The Influence of Divalent Metal Cations on the Nt.BspD6I Interaction with DNA

The impact of divalent metal ions on the activity of NEs from various organisms has not been evaluated so far. We hypothesised that Nt.BspD6I may share catalytic properties with both restriction endonucleases and other nicking endonucleases, such as bacterial proteins MutL from the MMR system and AP endonucleases. Although Mg^2+^ is the preferred cofactor for the majority of nucleases, Mn^2+^ also supports the DNA cleavage. Thus, the ability of Mn^2+^ and Co^2+^ in addition to magnesium ions to act as hydrolytic cofactors has been previously reported for R.EcoRV and R.EcoRI; in this context, the presence of Zn^2+^, Cd^2+^, Ni^2+^, or Ca^2+^ either strongly inhibits or eliminates the specific activities [27]. The relaxation of specificity termed ‘star activity’ [34] has been observed for R.EcoRI in the presence of Mn^2+^. We demonstrated that the efficiency of DNA cleavage by MutL from *Rhodobacter sphaeroides* decreases in the order Mn^2+^ > Co^2+^ > Mg^2+^ > Cd^2+^, whereas Ni^2+^, Ca^2+^, and Zn^2+^ do not stimulate its nicking activity [35]. Moreover, the active site of some type II restriction enzymes, e.g., R.KpnI, can accommodate not only Mn^2+^ but even Ca^2+^ and Zn^2+^ [36,37]. The effects of divalent metal ions (Mg^2+^, Mn^2+^, Ca^2+^, Zn^2+^, Cu^2+^, or Ni^2+^) on DNA binding and catalytic stages of AP endonuclease APE1 have been reported by us elsewhere [38]. It was shown in that study that the initial substrate binding, corresponding to the formation of an initial enzyme–substrate complex, does not depend on either the concentration or nature of divalent metal ions. At the same time, in the presence of a divalent ion, the enzymatic activity of APE1 decreased in the order Mg^2+^ > Mn^2+^ > Ni^2+^ > Zn^2+^. Nevertheless, APE1 hydrolysed substrate in the presence of 5 mM Zn^2+^. Cofactors’ concentrations of 5–10 mM are used in in vitro experiments in most cases.

**Figure 2:**
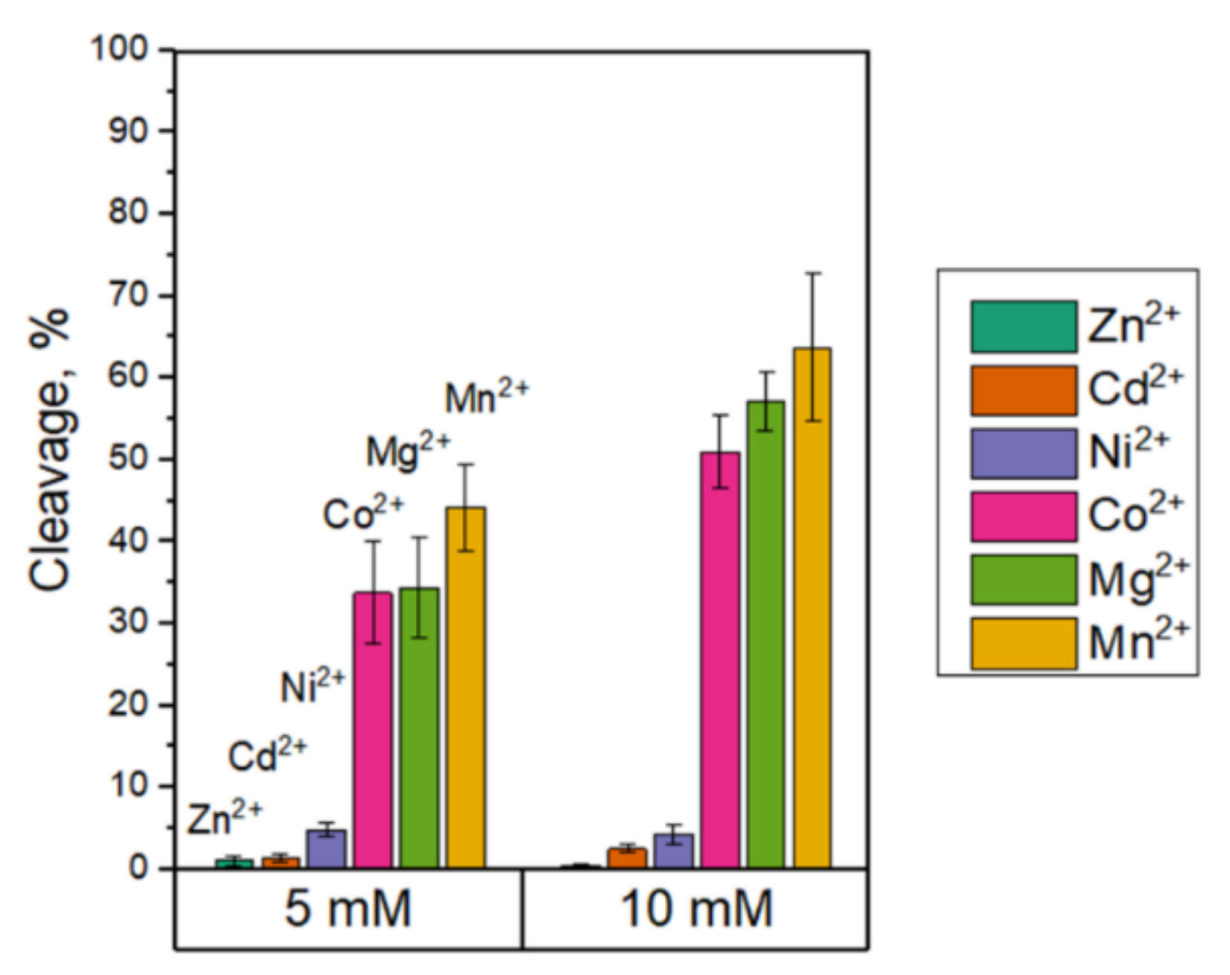
The influence of different divalent cations (at 5 or 10 mM) on the nicking activity of Nt.BspD6I. The nicking was not detectable in the presence of calcium ions.

Therefore, we assessed the efficiency of **VI-30** nicking by Nt.BspD6I during 30 min at 37 °C in the presence of one of divalent cations (5 and 10 mM) that presumably modulate the endonuclease activity: Mg^2+^, Mn^2+^, Ca^2+^, Co^2+^, Ni^2+^, Cd^2+^, or Zn^2+^ (Figure 2). Only Mn^2+^ and Co^2+^ are similar enough to Mg^2+^ to support effective Nt.BspD6I nicking activity. The same situation was observed in the case of 10 mM concentration of cofactors. In further experiments, we chose magnesium ions as the cofactor most commonly used for DNA phosphodiester bond hydrolysis [39,40] to examine the catalytic stage of the Nt.BspD6I interaction with DNA.

Ca^2+^ did not activate DNA nicking by Nt.BspD6I. We have demonstrated earlier that in the presence of Ca^2+^, Nt.BspD6I retains its ability to bind the target DNA sequence [10]. For REs, Ca^2+^ increases specific binding several-fold to >6000-fold relative to metal-free conditions ([39] and references therein). In the presence of Ca^2+^, the catalytic activity of APE1 disappears, with the retention of the binding ability [38]. Accordingly, Ca^2+^ is widely used in DNA-binding studies on many nucleases, and we employed it as well for characterising the formation of Nt.BspD6I–DNA complexes, including an unproductive enzyme–substrate complex.

### 3.3. Kinetics of Non-Specific Nt.BspD6I Binding to DNA

Many type II REs, e.g., R.EcoRI, R.BamHI, R.SmaI, and R.SfiI, bind more efficiently to a specific DNA region than to a non-specific one, even in the absence of Mg^2+^ [23,41,42]. In contrast, R.TaqI, R.Cfr9I, R.BcgI, R.EcoRV, and some others bind equally well to DNA with or without recognition sites [42,43,44,45]. On the other hand, for R.FokI, which is monomeric both in solution and when bound to DNA in the absence of divalent metal ions and has a similar structural organisation to Nt.BspD6I, the binding with non-specific DNA was not detected [46]. The information on the interaction of nicking endonucleases with DNA that does not contain the recognition site was not found. Thus, non-specific Nt.BspD6I binding to DNA was investigated in our work. 

It has been established by an electrophoretic mobility shift assay [47] that Nt.BspD6I has high affinity for non-specific DNA duplexes. It was shown in refs. [10,23] that the efficiency of the interaction of REs or Nt.BspD6I with DNA depends on the length of the duplex. At the same time, *K_d_* values of Nt.BspD6I complexes with 26 bp or 30 bp substrates characterise the sum of specific and non-specific binding and are comparable (7 ± 1 and 8 ± 1 nM, respectively) [10]. Thus, the binding site of Nt.BspD6I in DNA does not exceed 26 bp. Accordingly, to increase the signal-to-noise ratio in the kinetic curves, in the assays of the Nt.BspD6I non-specific binding, we selected a 30 bp DNA duplex, **VI-30N**, which does not contain the enzyme’s recognition site. In control experiments, we used the **VI-30** DNA duplex, which contains Nt.BspD6I’s recognition site (Table 1).

**Figure 3:**
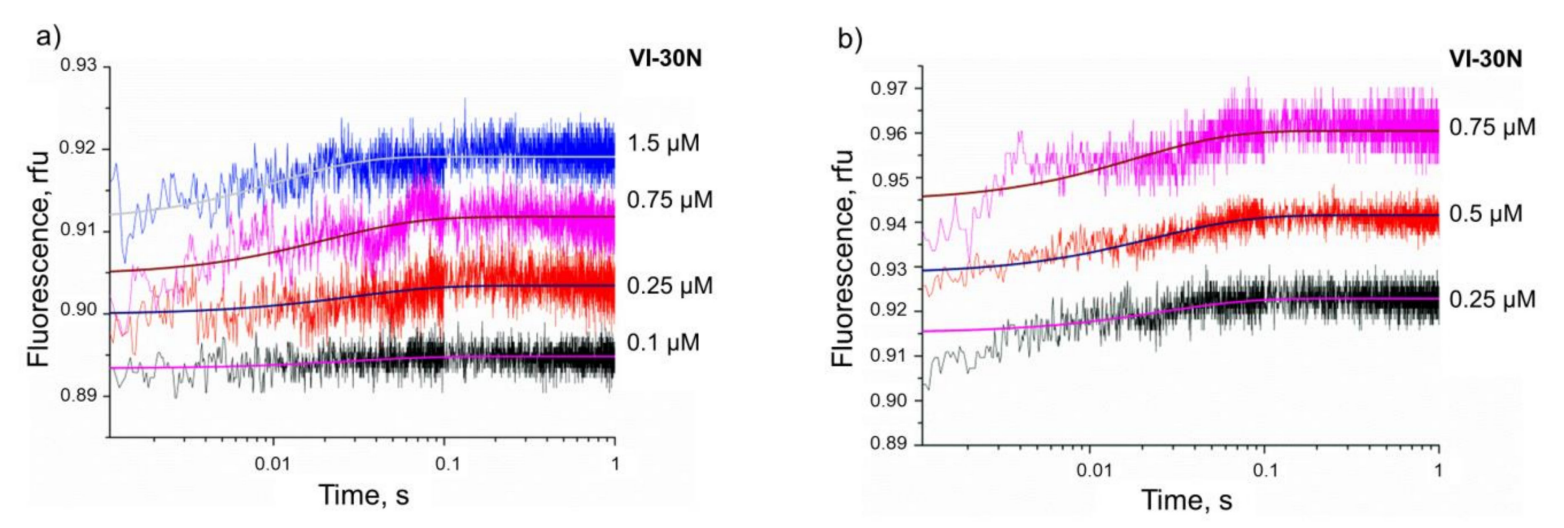
Kinetic curves characterising the interaction of Nt.BspD6I (1 μM) with non-specific DNA duplex **VI-30N** (concentrations are shown next to the curves): in the presence of (**a**) 10 mM CaCl_2_ or (**b**) 10 mM MgCl_2_. Coloured dots indicate experimental data, and solid curves denote calculated kinetic curves corresponding to Scheme 1. Rfu: relative fluorescence units.

To investigate the process of non-specific binding of Nt.BspD6I to DNA, we used the stopped-flow method with detection of conformational changes in the enzyme. We assumed that during the formation of a non-specific complex, only insignificant conformational changes take place in the protein molecule, and as a consequence, only a small change in the fluorescence intensity can be expected. A series of the kinetic curves was constructed for the Nt.BspD6I interaction with various concentrations (0.1–1.5 μM) of DNA duplex **VI-30N** in the presence of either 10 mM CaCl_2_ or 10 mM MgCl_2_ (Figure 3). The reactions were allowed to proceed at 37 °C, and the enzyme concentration was identical among these assays (1 μM).

An analysis of the kinetic curves suggested that the Nt.BspD6I interaction with a non-specific 30 bp DNA duplex in the presence of either Ca^2+^ or Mg^2+^ is described by kinetic Scheme 1, which includes one reversible stage of complex formation between the enzyme and DNA.

Kinetic rate constants of elementary reaction stages (*k*_1_ and *k*_−1_) were optimised in the DynaFit software [18] by the method of numerical integration of a system of differential equations in accordance with Scheme 1. The rate constants and dissociation constants of the Nt.BspD6I–DNA complex calculated from the formula *K_d_* = *k_−_*_1_/*k*_1_ are given in Table 2. It should be noted that the kinetic and thermodynamic parameters of Scheme 1 were virtually the same regardless of whether the reaction mixture contained Ca^2+^ or Mg^2+^. In other words, the nature of the divalent metal ion in this case did not affect the formation of a non-specific complex between Nt.BspD6I and DNA. Of note, in the case of R.EcoRV, Ca^2+^ and Mg^2+^ stimulate the binding to a specific or non-specific DNA substrate approximately equally [48,49].

**Figure 4:**
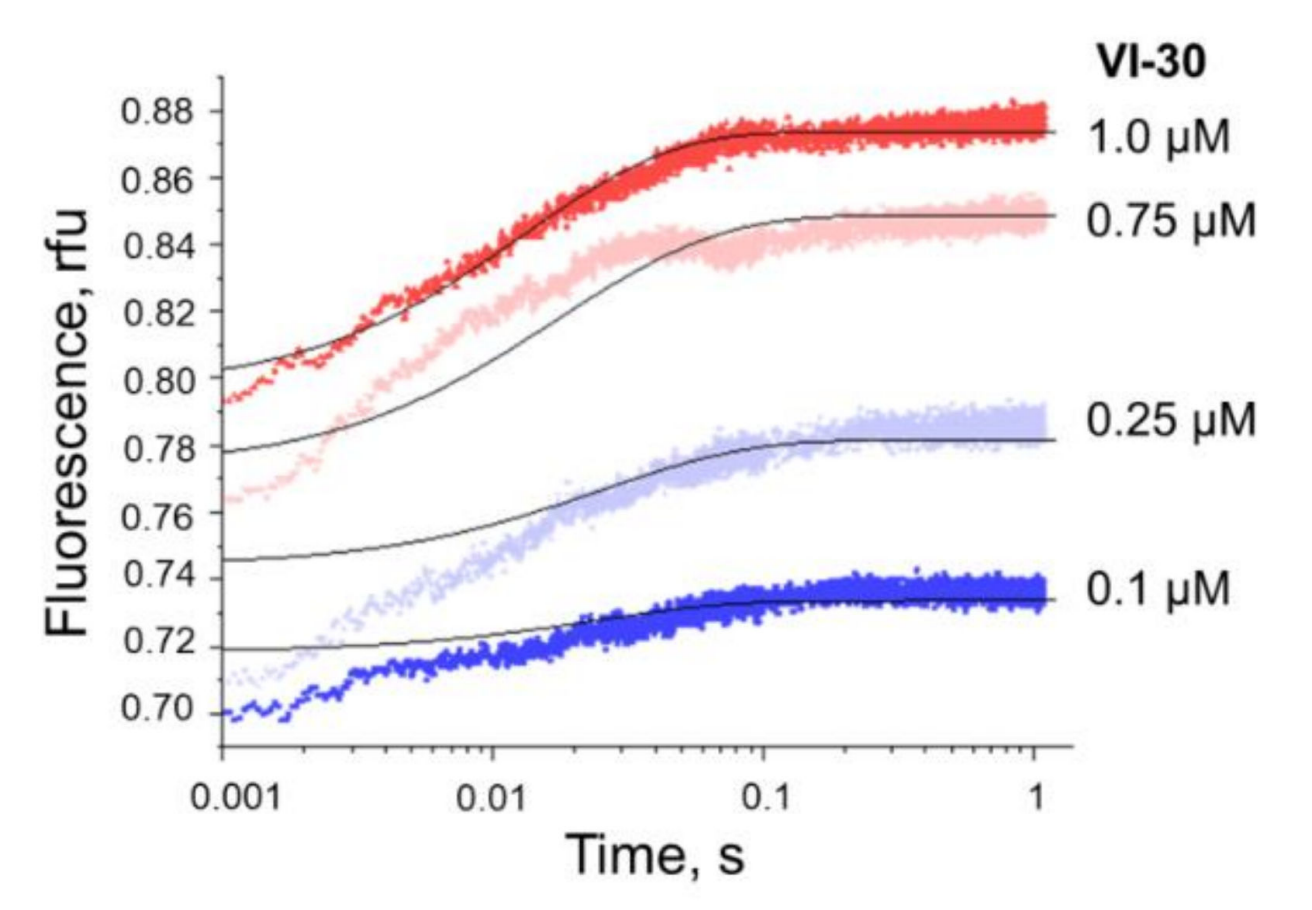
Kinetic curves for the interaction of Nt.BspD6I (1 μM) with DNA duplex **VI-30**, which contains the enzyme’s recognition site. The measurements were performed in the absence of divalent metal ions; duplex concentrations are shown next to the curves. Coloured dots indicate experimental data, and solid curves denote calculated kinetic curves corresponding to Scheme 1, which contains one reversible stage of complex formation.

To assess the influence of the Nt.BspD6I recognition site on the efficiency of protein–nucleic acid complex formation in the absence of divalent metal ions, a series of kinetic curves for the binding of 30 bp DNA duplex **VI-30** with the NE was obtained (Figure 4). The reactions were carried out under the same conditions as in the case of duplex **VI-30N**. EDTA was added to the buffer to a concentration of 0.4 mM.

The interaction of Nt.BspD6I with duplex **VI-30** in these settings is also characterised by one reversible stage of complex formation (Scheme 1). It should be mentioned that the addition of a reversible stage to Scheme 1 did not increase the accuracy of the fit to the experimental data (Figure S2). Therefore, the one-step model was chosen as the final one; the reaction parameters are presented in Table 2. The rate constant for the formation of the Nt.BspD6I complex with duplex **VI-30** (containing the recognition site), *k*_1_, is approximately 30% higher than the corresponding rate constants for the Nt.BspD6I binding to duplex **VI-30N**, and the dissociation constant *K_d_* of the complex is ≈five-fold lower, even in the absence of divalent metal ions. Nevertheless, in this case, the difference in ionic strength between the buffers could also have some influence on the enzyme function. Therefore, the initial interaction of Nt.BspD6I with DNA does not require divalent metal ions. Such a fact was also observed for endonucleases MutH and HinP1I that make the nick in dsDNA under certain conditions [50,51] as well as for monomeric two-domain R.FokI [46]. Nevertheless, just as many type II REs Nt.BspD6I bind more efficiently to specific DNA than to non-specific DNA, even in the absence of divalent metal ions. In contrast to R.FokI, DNA cleavage reaction by Nt.BspD6I proceeds without the enzyme dimerisation step [8,52].

### 3.4. Formation of a Complex between Nt.BspD6I and DNA Substrates of Different Lengths

The formation of a specific DNA–protein complex gives rise to numerous contacts with heterocyclic bases (a special pattern of hydrogen bonds) and electrostatic and hydrophobic contacts with the sugar-phosphate backbone of the recognition site [53]. By contrast, in many studies on RE–DNA complexes performed by X-ray and chemical footprinting, it has been demonstrated that non-specific contacts of the enzyme with DNA are outside the recognition site, i.e., in the regions flanking it. For the effective functioning of many type II REs, a 2–4 bp sequence flanking the recognition site is necessary. It is believed that such additional interactions play an important role in the stabilisation of a specific catalytically competent complex [23].

It has been reported earlier that for the Nt.BspD6I enzymatic reaction to proceed, the flanking sequence on the 5′ side of the recognition site should be at least 2 bp, while on the 3′ side of the strand cleavage site, the flanking sequence must be at least 3 bp [10]. The influence of the length of flanking sequences on the kinetics of complex formation between Nt.BspD6I and a DNA substrate has not been researched to date.

We constructed a set of DNA substrates (**I-17**, **II-19**, **III-21**, **IV-23**, **V-26,** and **VI-30**) 17 to 30 bp in length, where the number of base pairs downstream of the cleavage site (in the 3′ direction) ranges from 2 to 11 (Table 1). DNA duplexes **IV-23** and **V-26** contain the same (8 bp) number of base pairs downstream of the cleavage site, but **V-26** contains an additional 3 bp on the 5′ side of the recognition site. As compared to **V-26**, the DNA duplex **VI-30** is extended by 1 bp on the 5′ side of the recognition site and by 3 bp downstream of the cleavage site.

**Figure 5:**
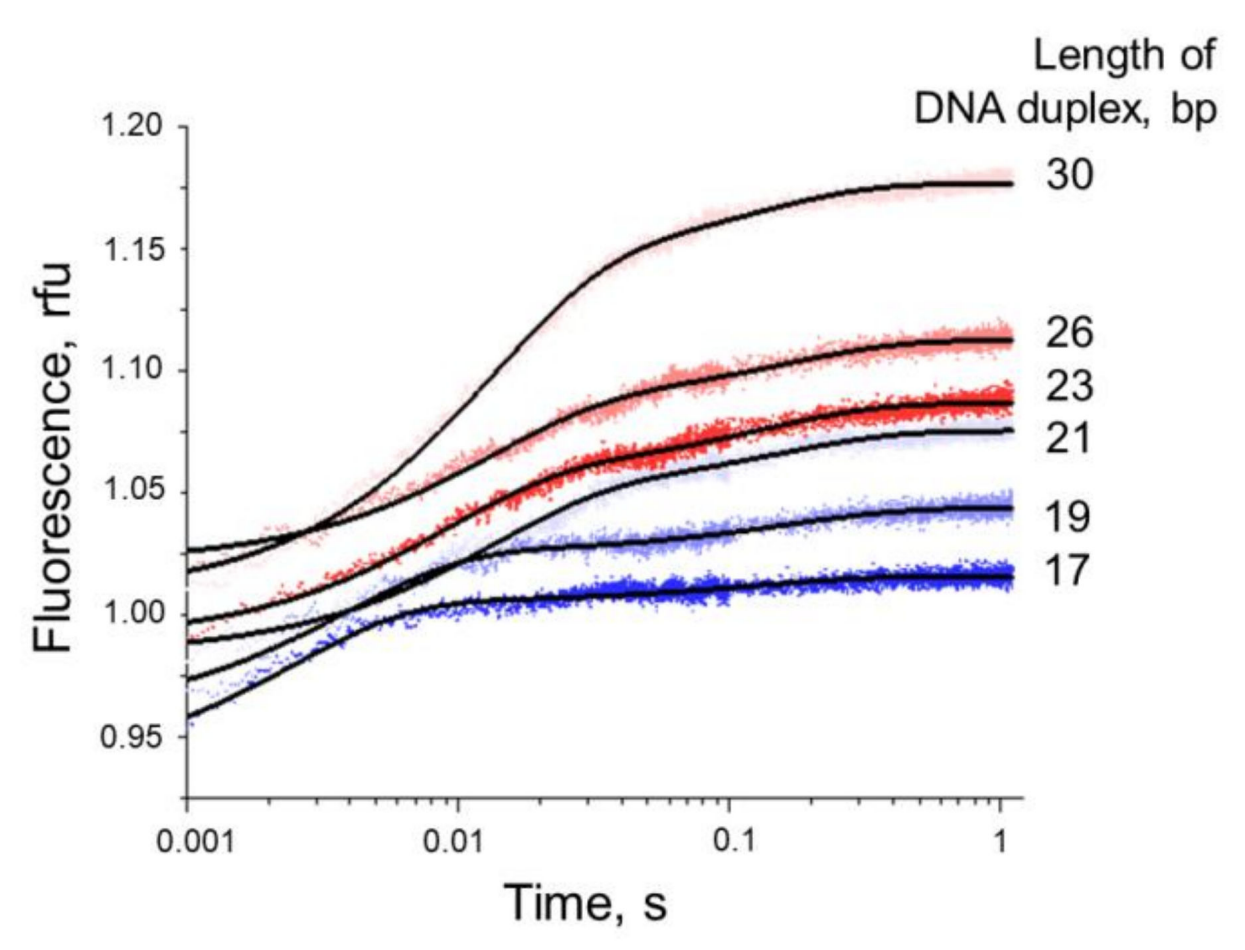
Kinetic curves characterising the interaction of Nt.BspD6I (1 μM; in the presence of 10 mM CaCl_2_) with DNA duplexes (1 μM) of various lengths (indicated to the right of the curves). The coloured dots denote the experimental data, and each solid curve represents their fit to the equation $y=C+A_{1}e^{-k_{1}x}+A_{2}\left(1-e^{-k_{2}x}\right)$ (Table S2). In the presence of 10 mM MgCl_2_, similar curves were obtained (Figure S3).

The conformational alterations in the enzyme molecule during the reaction were monitored via changes in the fluorescence of Nt.BspD6I Trp residues in the free state and in the complex with DNA. The kinetic curves were recorded between time points 10 ms and 1 s in the presence of either Ca^2+^ or Mg^2+^. According to ref. [10], in the 1 s range, cleavage does not occur at all or is just beginning. In addition, both series of kinetic curves have the same pattern of changes in Trp fluorescence intensity (Figure 5); therefore, it is possible that in the presence of either Ca^2+^ or Mg^2+^, only the binding was monitored in the interval between 0 and 1 s.

By the non-linear regression method in the Origin software, the kinetic curves of the Nt.BspD6I interaction with each of the duplexes were fitted to the Equation (1).

**Scheme 3:**
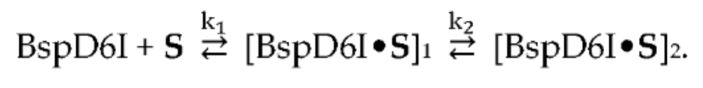
The kinetic schemes of the biochemical reactions 3.

This interaction is characterised by two exponents, which correspond to the number of stages in the reaction mechanism (Figure 5). Accordingly, the observed transformations can be represented by two-stage Scheme 3.

Examination of the rate constants observed for the Nt.BspD6I interaction with DNA duplex **I-17**, **II-19**, **III-21**, **IV-23**, **V-26**, or **VI-30** revealed that *k*_1_ significantly decreases with the increasing length of the DNA duplexes in the presence of either Ca^2+^ or Mg^2+^ (Table 3).

**Figure 6:**
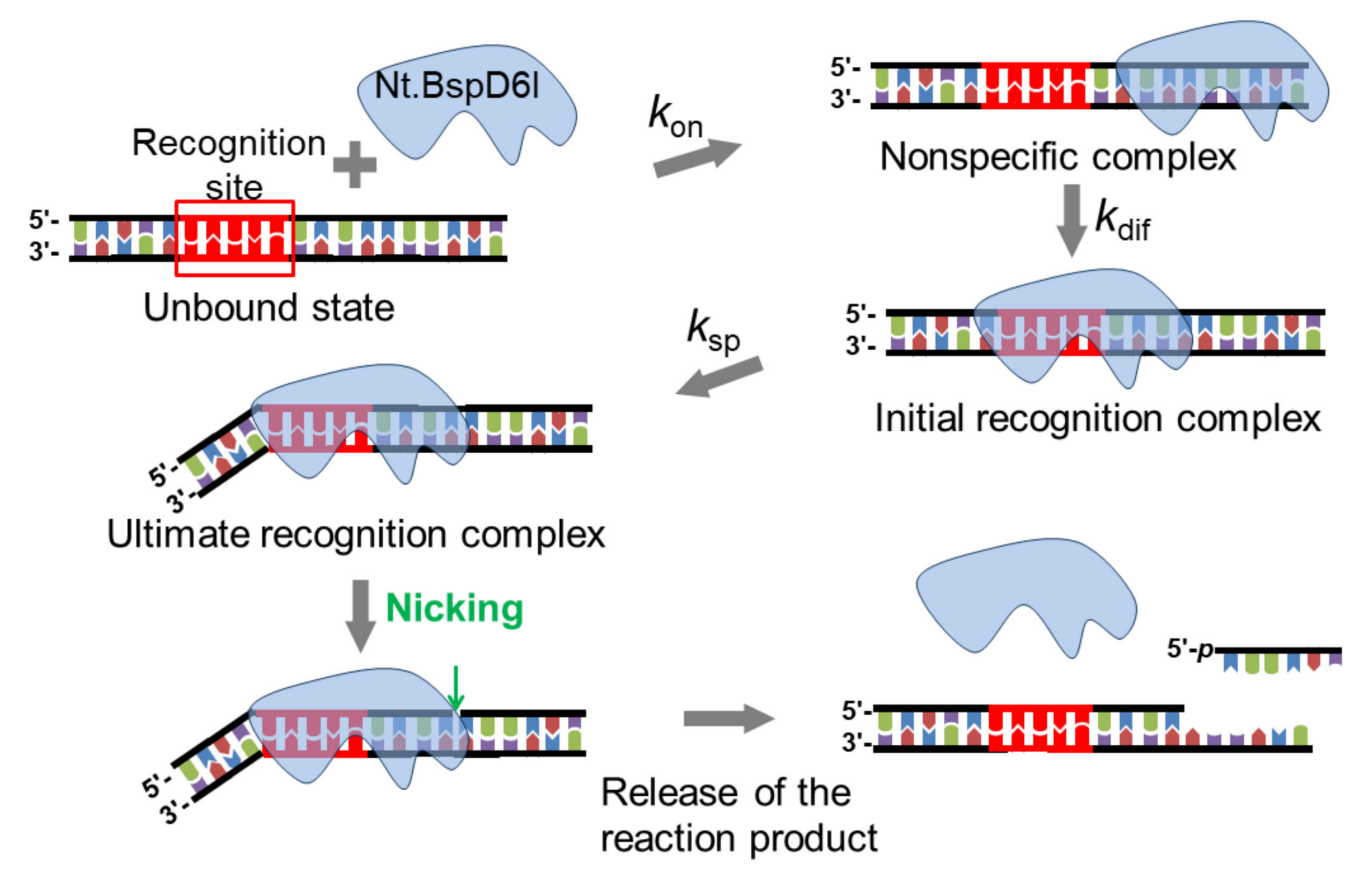
An outline of the mechanism behind the interaction of Nt.BspD6I with a substrate. *k_on_*: The rate constant for the formation of a non-specific complex, *k_dif_*: the rate constant for the search for a target sequence, *k_sp_*: the rate constant for the formation of specific contacts between the protein and DNA. The protein recognition site is red.

It can be assumed that the rates of the emergence of a non-specific enzyme–substrate complex (and the formation of specific contacts between the enzyme and target sequence 5′-GAGTC-3′/5′-GACTC-3′) should not depend on the total length of the DNA duplex. Therefore, the main contribution to the dependence of the observed binding rate constant k_1_ on the DNA duplex length is made by the stage of appearance of temporary non-specific contacts between Nt.BspD6I and the sequences flanking the enzyme’s recognition site during the search for the latter. After non-specific binding, the enzyme continuously moves along the DNA in search of structural elements characteristic of its recognition site; this process is called linear diffusion [23] (Figure 6). In the case of long DNA substrates of REs, for example, plasmid or phage DNA, both the sliding of the enzyme along the DNA and diffusion of the ‘hopping and jumping’ type are expected; the latter implies a microscopic dissociation–association of a non-specific protein–nucleic acid complex and ‘jumping’ of the enzyme by several base pairs [54,55,56]. When the length of the substrate is approximately equal to the binding site of the enzyme ‘landing’ on the DNA, the most probable mechanism of diffusion is ‘hopping and jumping’.

Based on all of the above, it is likely that the first rate constant k_1_ indicated in Scheme 3 characterises the processes of Nt.BspD6I non-specific binding to DNA and its subsequent movement along DNA in search of a specific sequence.

**Figure 7:**
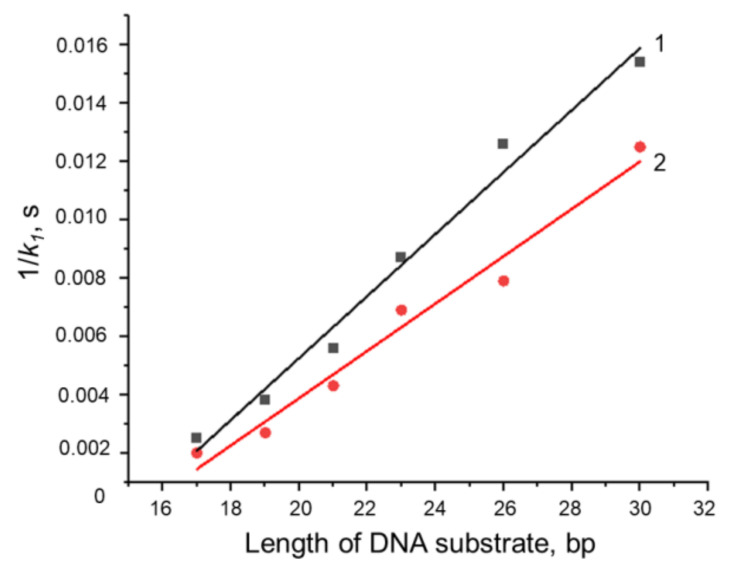
Dependence of characteristic time (τ = 1/*k*_1_) of the Nt.BspD6I binding to the target sequence on the length of the DNA duplex in the presence of Ca^2+^ (**1**) or Mg^2+^ (**2**). The dots denote the experimental data, and the curves represent their fit to the equation *1*/*k*_1_ = *1*/*k_on_* + *1*/*k_dif_* × n, where n is the length of the DNA duplex (bp).

The characteristic time (a reciprocal of the observed rate constant: τ_1_ = 1/*k*_1_) [57,58] required for the Nt.BspD6I binding to the 5′-GAGTC-3′/5′-GACTC-3′ sequence increases linearly with the length of a DNA duplex (Figure 7). To note, in the case of 1D linear diffusion in a random direction, the average search time is proportional to (length)^2^ [59,60]. Since *k*_1_ was obtained for substrates with a narrow range of length, the linear dependence of 1/*k*_1_ was found, which represents a part of the total quadratic dependence expected for a wide length range.

The observed rate constant (*k_on_*) for the assembly of a non-specific complex can be estimated from k_1_ for a DNA duplex with minimum length (for **I-17**, *k_on_* = *k*_1_). In the case of movement of the enzyme from the end of this duplex towards the recognition site (Figure 7), the contribution of the diffusion to the search for a specific sequence can be ignored because only 6 bp separate the recognition site from both termini of this DNA duplex. On the other hand, with an increase in the number of base pairs flanking the DNA cleavage site (as in the series **II-19**, **III-21**, **IV-23**, **V-26** and **VI-30**), the contribution of the diffusion stage enlarges and can be described by the slope of the straight line corresponding to search time (*τ_dif_* = 1/*k_dif_*):

(4) $$\tau_{1}=\tau_{\mathrm{on}}+\tau_{\mathrm{dif}}\times n$$

or

(5) $$\frac{1}{k_{1}}=\frac{1}{k_{\mathrm{on}}}+\frac{1}{k_{\mathrm{dif}}}\times n$$

where n is the length of the DNA duplex in bp.

By means of this equation, *k_on_* and *k_dif_* were computed for the interaction of Nt.BspD6I with DNA duplexes in the presence of either Ca^2+^ or Mg^2+^ (Figure 7). Observed rate constants *k_on_* for the formation of a non-specific complex are roughly similar when compared between the ions (500 and 400 s^−1^ for the reactions involving Mg^2+^ and Ca^2+^, respectively). It is worth noting that diffusion rate constant *k_dif_* depends on the nature of the metal ion and is 1400 and 880 s^−1^ for Mg^2+^ and Ca^2+^, respectively. Thus, in the presence of the hydrolytic-reaction cofactor, Mg^2+^, Nt.BspD6I searches for a specific nucleotide sequence almost 1.5-fold faster. Adding several base pairs only on the 5′ side (as in **II-19**, **III-21**, and **IV-23**), only on the 3′ side (switching from **IV-23** to **V-26**), or on both sides (switching from **V-26** to **VI-30**) of the substrate recognition site does not disrupt the linearity of the dependence of characteristic time τ_1_ on DNA length. In other words, *k_dif_* does not depend on the sequence and remains constant as the length of the substrate increases on both the 3′ and 5′ side.

The *k*_2_ constant does not depend on the DNA length in the presence of Ca^2+^. On the contrary, in the presence of Mg^2+^, the formation of the complex slows down with the extension (from 2 to 8 bp) of the region flanking the site of nicking on the 3′ side, and then, the rate of the process stabilises (Figure 7). Therefore, we believe that the second observed rate constant *k*_2_ matches *k_sp_* in Figure 6 and characterises the stage of assembly of the final recognition complex; this stage includes the emergence of all specific contacts with the recognition site and complete DNA bending required for the hydrolysis of the phosphodiester bond. It turned out that the shorter the DNA duplex, the faster this process. With an increase in DNA duplex length to 23 bp (**IV-23**) and further (**V-26** and **VI-30**), rate constants *k*_2_ do not change further. Therefore, either an increase of 3 and 4 bp in the sequence flanking the recognition site on the 5′ side (switching from **IV-23** to **V-26** and then to **VI-30**) or an increase of 3 bp in the sequence flanking the site of incision on the 3′ side (switching from **V-26** to **VI-30**) does not affect the formation kinetics of the final recognition complex. We can conclude that in the duplex, Nt.BspD6I interacts with at least 8 bp downstream of the cleavage site (12 bp downstream of the recognition site) and with 4 bp upstream of the recognition site, as reported earlier [10]. Our data indicate that the binding site of Nt.BspD6I in DNA is at least 21 bp.

### 3.5. Kinetics of Specific Interaction of Nt.BspD6I with a DNA Substrate

The final process that defines the Nt.BspD6I function is the finding of its recognition site in DNA, the formation of a specific complex, and nicking of the DNA substrate. We characterised this process in detail using two approaches: the analysis of the substrate cleavage by Nt.BspD6I under steady-state conditions and the assay of conformational dynamics of DNA substrates when interacting with Nt.BspD6I in the presence of either Ca^2+^ or Mg^2+^.

#### 3.5.1. Steady-State Kinetics of Nt.BspD6I Interaction with DNA

**Figure 8:**
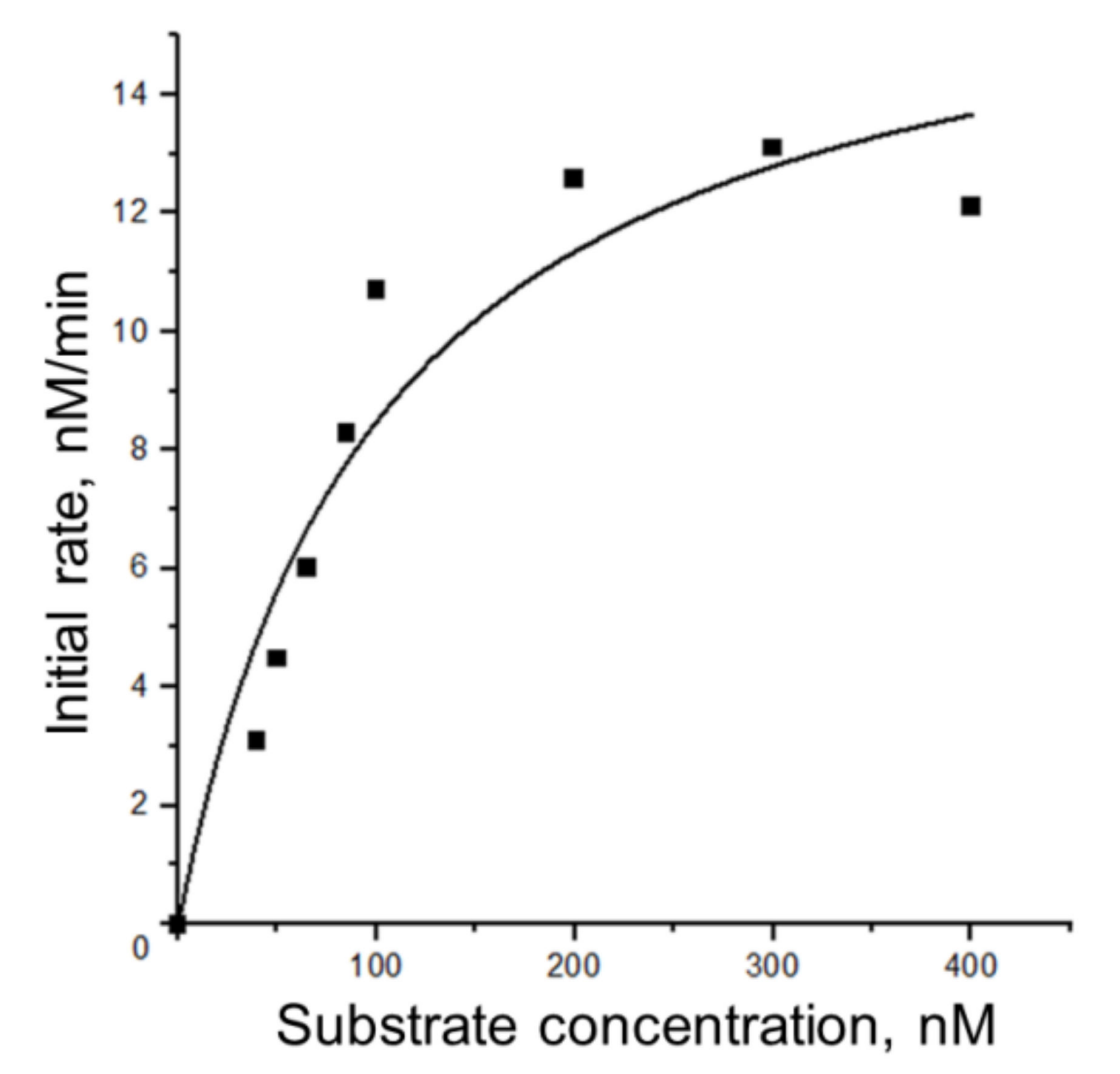
Dependence of the initial rate of **II-19A** nicking by Nt.BspD6I (7 nM) on the DNA substrate concentration. Each data point is an average from at least three independent experiments.

The kinetics of the Nt.BspD6I reaction with a 19 bp DNA duplex **II-19A** containing a fluorescent FAM tag at the 5′ end of the top strand, in which an internucleotide bond is hydrolysed in the presence of Mg^2+^ ions, were assayed as described in detail in Section 2.3 (Figure 8, Figures S4 and S5, Scheme S1). The obtained values are comparable with the literature data on type II REs with similar substrates (Table 4).

The introduction of reporter molecules at the ends of a DNA duplex can affect the efficiency and kinetics of its interaction with Nt.BspD6I. A control experiment was conducted, too: steady-state kinetics of Nt.BspD6I-catalysed nicking of 19 bp DNA duplexes **II-19** and **II-19B**, which were ^32^P-labelled at the 5′ end of the top strand, were compared with steady-state kinetics of **II-19A** nicking under the same conditions. The half-transformation time of the substrate for all three DNA duplexes was 8 ± 1 min (data not shown). Therefore, in this context, the presence of FAM and BHQ1 does not affect the kinetics of DNA nicking by Nt.BspD6I.

#### 3.5.2. Conformational Dynamics of DNA Substrates during Interaction with Nt.BspD6I

To register conformational alterations of DNA substrates during interaction with Nt.BspD6I, 19 bp DNA substrates **II-19C** and **II-19D** containing the FAM/BHQ1 pair of dyes were used (Table 1). On the one hand, such 19 bp DNA duplexes contain enough base pairs flanking the enzyme recognition site and the incision position that are necessary for the efficient functioning of Nt.BspD6I [10]. On the other hand, the distance between the FAM and BHQ1 groups (≈65 Å for **II-19C**) is sufficient for effective energy transfer between them [63].

DNA duplex **II-19C** contains FAM at the 5′ end and BHQ1 at the 3′ end of the top strand. We assumed that such an arrangement of dyes would make it possible to detect DNA bending by means of a decrease in the fluorescence intensity during complex formation with Nt.BspD6I owing to the approach of the FAM fluorophore to the BHQ1 fluorescence quencher (Figure S5a). DNA duplex **II-19D** served as a control: it contains FAM at the 3′ end of the top strand and BHQ1 at the 5′ end of the bottom strand. In this assay, DNA bending during the complex formation presumably did not affect the distance between the dyes and therefore would not lead to significant changes in the fluorescence intensity (Figure S5b). The dissociation of the DNA nicking product should cause an increase in the fluorescence intensity for both duplexes owing to a greater distance between the reporter groups (Figure S5c,d).

**Figure 9:**
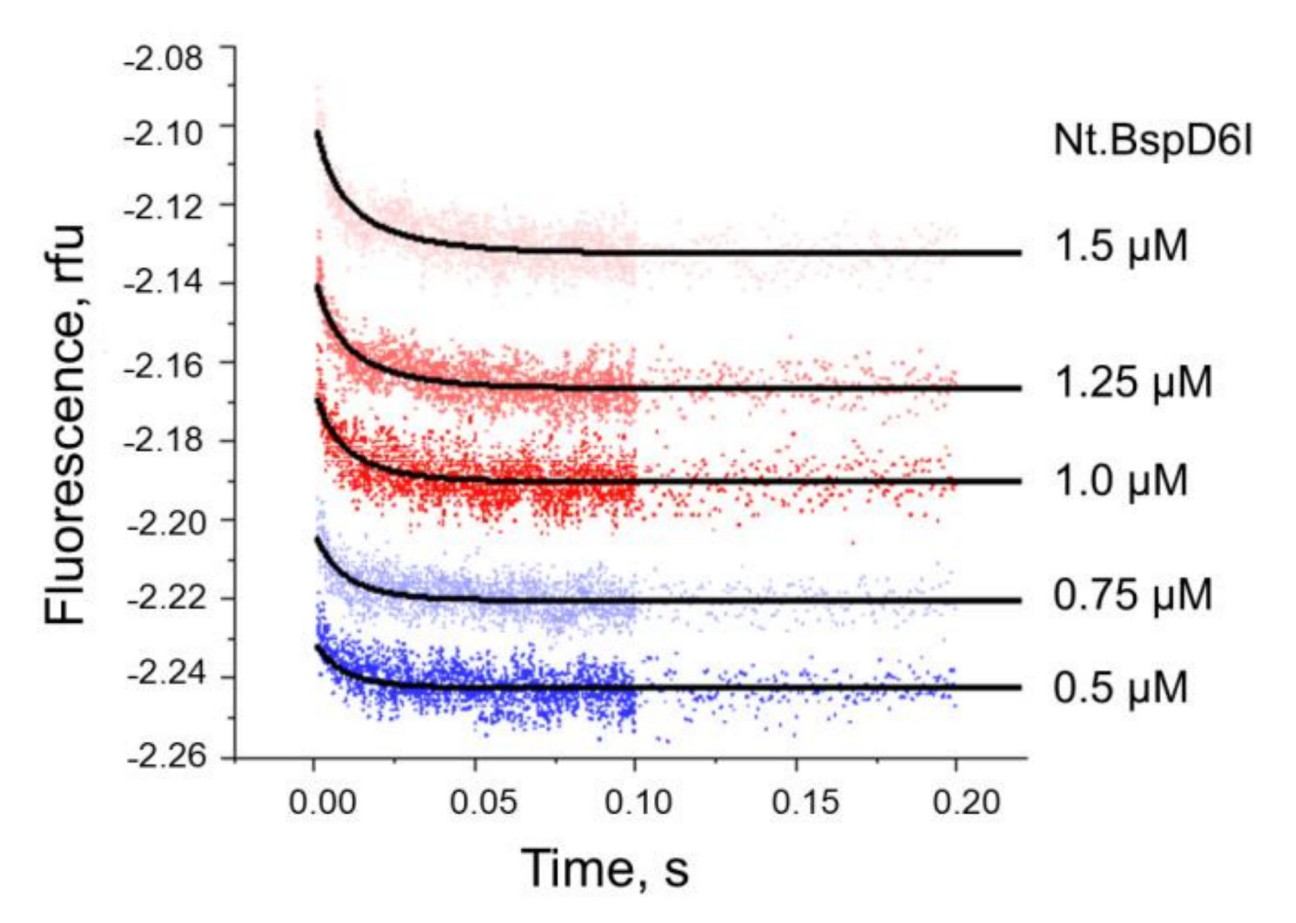
Kinetic curves for the interaction of Nt.BspD6I (concentration is indicated to the right of the curves) with 1 µM duplex **II-19C** in the presence of 10 mM CaCl_2_. Coloured dots indicate experimental data, and solid curves denote calculated kinetic curves conforming to Scheme 1.

Initially, the process of Nt.BspD6I binding to the two substrates was assessed in the presence of 10 mM CaCl_2_. A series of kinetic curves was built for the formation of an unproductive enzyme–substrate complex. We tested 0.25–1.50 µM Nt.BspD6I solutions at a fixed concentration of the DNA substrate (1 µM; Figure 9). In the presence of Ca^2+^, the interaction of Nt.BspD6I with DNA duplex **II-19C** (containing FAM at the 5′ end and BHQ1 at the 3′ end of the ‘top’ strand) resulted in a one-phase decline of fluorescence intensity (Figure 9). This finding points to a decrease in the distance between the ends of the duplex in the complex with the enzyme and confirms the bending of the DNA substrate by the enzyme during the complex formation. An analysis of the kinetic curves suggested that Scheme 1 (Section 2.5), containing one reversible stage, satisfactorily describes the experimental curves. On the other hand, due to the low signal-to-noise ratio, the rate constants were determined with a large error (Table 5). Nevertheless, the dissociation constant *K_d_* of the enzyme–substrate complex (9 ± 7 nM) was consistent with the *K_d_* obtained earlier in an electrophoretic mobility shift assay (7–8 nM) [10].

Thus, our experiments confirmed the data generated by the method of circular permutations in ref. [10], suggesting that the stage of Nt.BspD6I specific binding to the recognised nucleotide sequence is characterised by a DNA bend, which was estimated to be 66° ± 4°.

**Figure 10:**
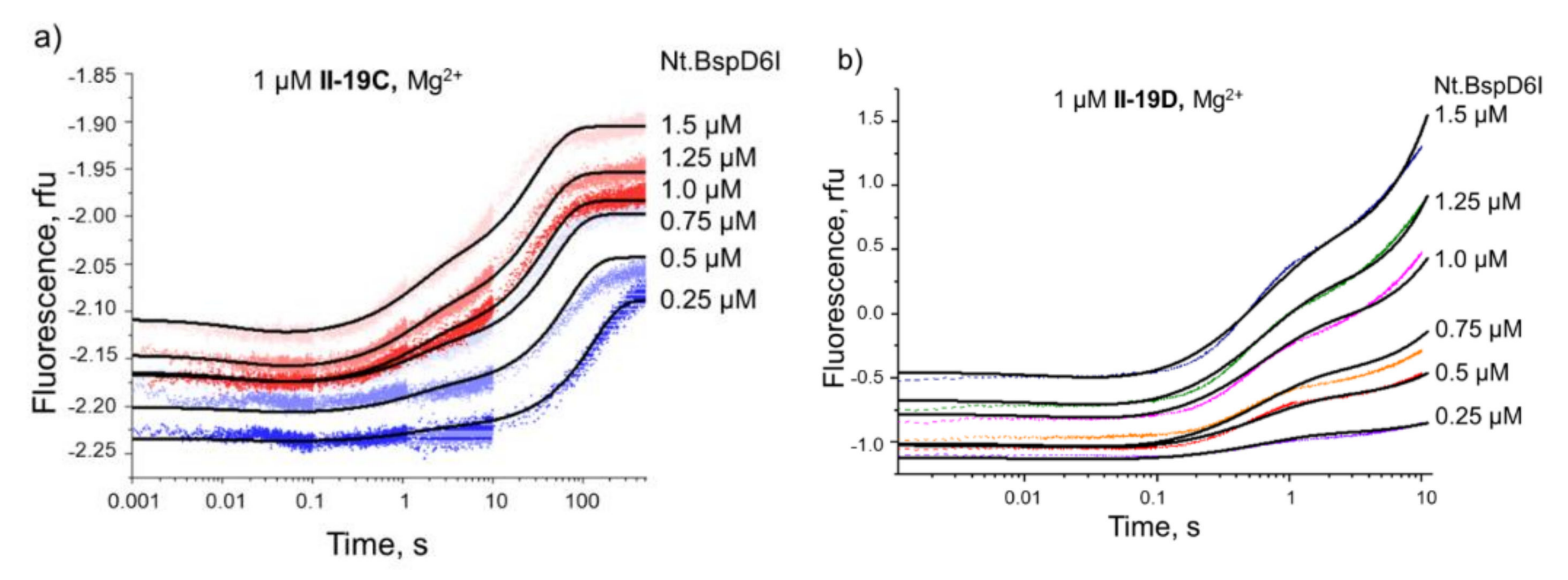
Kinetic curves describing the interaction of Nt.BspD6I (concentration is indicated on the right) in the presence of 10 mM MgCl_2_ either (**a**) with 1 µM duplex **II-19C** or (**b**) with 1 µM duplex **II-19D**. Coloured dots and lines indicate experimental data, and black curves denote calculated kinetic curves conforming to Scheme 2.

Next, we evaluated interaction kinetics of the same 19 bp DNA substrates with Nt.BspD6I in the presence of 10 mM MgCl_2_, i.e., when the cleavage of both substrates was possible. We employed 0.25–1.50 μM enzyme solutions at a constant DNA substrate concentration of 1 μM (Figure 10). For **II-19C**, in the presence of Mg^2+^ ions, the formation of the enzyme–substrate complex was accompanied by a slight decline of the fluorescence intensity at time points up to 100 ms (Figure 10a) owing to the approach of the FAM fluorophore to the BHQ1 fluorescence quencher upon DNA bending (Figure 6). Then, there was a two-phase increase in the fluorescence intensity until time points 100–500 s for various concentrations of the enzyme. The first phase of signal growth in the kinetic curves probably matches the progress of the DNA phosphodiester bond hydrolysis, and the second phase coincides with the release of the product of this reaction from its complex with the enzyme. Note that at time points up to 1 s, the hydrolysis almost does not take place (for lower enzyme concentrations) or only begins (for higher enzyme concentrations). These findings confirm our assumption made in Section 3.4.

Therefore, the minimal kinetic scheme of the interaction of specific duplex **II-19C** with Nt.BspD6I, as detected by means of conformational transformations of the substrate, should include three stages: reversible formation of the enzyme–substrate complex, irreversible DNA nicking, and reversible dissociation of the enzyme from the reaction product (Scheme 2). Moreover, the product release stage could actually include a few different processes such as enzyme reorganisation after substrate cleavage, partial dissociation of a short oligonucleotide fragment from the enzyme–product complex owing to melting of the 3′-terminal part of the nicked DNA, and finally complete dissociation of the enzyme and DNA product.

With Ca^2+^ ions, the interaction of Nt.BspD6I with the DNA duplex **II-19D** (containing FAM at the 3′ end of the ‘top’ strand and BHQ1 at the 5′ end of the ‘bottom’ strand, i.e., at the same terminus of the DNA duplex but in different DNA strands) did not lead to a noticeable change in the fluorescence intensity (Figure S6). As expected, the stage of complex formation for such a DNA substrate was not detectable, because there was no change in the distance between the fluorophore and fluorescence quencher. During the Nt.BspD6I interaction with duplex **II-19D** in the presence of Mg^2+^ (Figure 10b), the formation of the enzyme–substrate complex also does not concur with a significant change in the distance between the FAM fluorophore and BHQ1 fluorescence quencher. Therefore, there is no alteration of the fluorescence intensity in the kinetic curves at time points ≤100 ms. 

In contrast, the irreversible stage of DNA nicking and the stage of the reaction product release for duplex **II-19D** were accompanied by a biphasic increase in the fluorescence intensity. The DynaFit software was used to optimise the kinetic rate constants of the elementary steps included in Scheme 2 and describing the interaction of Nt.BspD6I with both types of substrate (Table 5). Rate constant *k*_2_ in this scheme matched the catalytic reaction rate constant in the Michaelis–Menten scheme (*k*_2_ = *k_cat_*), and the Michaelis constant was computed via the formula *K_M_* = (*k*_2_ + *k_–_*_1_)/*k*_1_. It must be pointed out that the kinetic parameters obtained by the stopped-flow method (Table 5) were in agreement with the steady-state kinetic data (Table 4).

As follows from Table 5, the formation of the Nt.BspD6I complex with **II-19C** has identical rate constants *k*_1_ in the presence of either Mg^2+^ or Ca^2+^. By contrast, inverse rate constant *k_–_*_1_ is almost 20-fold higher in the assay with Mg^2+^ ions, thereby increasing the dissociation constant of this complex. It can be theorised that such destabilisation of the complex by Mg^2+^ accelerates the search for the Nt.BspD6I recognition site in DNA, consistently with the increased rate constant of linear diffusion along the DNA towards the target sequence, *k_dif_* (Figure 6 and Figure 7). It should be noted that for model duplex **II-19D**, which does not allow us to register the process of DNA bending, the rate constants for the formation of the enzyme–substrate complex are lower in comparison with duplex **II-19C** (Table 5). Nonetheless, dissociation constants of complexes with Nt.BspD6I are almost identical for **II-19C** and **II-19D** in the presence of Mg^2+^ ions.

Thus, Nt.BspD6I bends specific DNA during complex formation in the presence of either Ca^2+^ or Mg^2+^; however, with Ca^2+^, the enzyme–substrate complex is unproductive. In the presence of Mg^2+^, the DNA bending process precedes the irreversible stage of DNA cleavage and the reversible release of the reaction product from its complex with the enzyme (Scheme 2). It is noteworthy that the emergence of a DNA bend upon binding to a specific sequence is not typical for all type II REs: in complex with R.FokI, which is known to be structurally similar to Nt.BspD6I, the DNA duplex remains in the canonical form [53]. R.EcoRI and R.EcoRV bend DNA upon binding to a specific sequence, but the bending angle is 30° and 50°, respectively, which is significantly less than that for Nt.BspD6I [10].

## 4. Conclusions

Summarising all the results, we propose a molecular mechanism of Nt.BspD6I action (Figure 6). At the first stage, initial non-specific binding of the enzyme to DNA occurs, which does not require the presence of a cofactor (bivalent metal ions) in the reaction mixture, as demonstrated using 30 bp DNA duplexes during the assay of Trp fluorescence. The stage of searching for the recognition site probably follows, which is accompanied by the formation of temporary contacts with the duplex sequences flanking Nt.BspD6I’s specific recognition site. The rate of this process was shown to depend on the nature of the cofactor and to increase 1.5-fold in the presence of Mg^2+^ in comparison with Ca^2+^. At the next stage, Nt.BspD6I makes specific contacts with the recognition site and non-specific contacts with at least 8 bp in the sequence flanking the strand cleavage site on the 3′ side in the substrate. As a consequence of these interactions, DNA bending takes place. After the final recognition complex of Nt.BspD6I with DNA is assembled in the presence of Mg^2+^ ions, there is an irreversible stage of substrate nicking and dissociation of the enzyme–product complex. Note that these stages are separated in time, which is in line with previously published data on the ability of Nt.BspD6I to form a complex with the reaction product [10].

One of the successful applications of NEs in biotechnology is the detection of nucleic acid sequences and even different base modifications [3]. The key stage of these techniques is hybridisation of the DNA to be detected with a fluorescently labelled DNA probe. In this context, it is necessary that single-stranded fragments of the NE’s recognition site be included in the nucleotide sequences of both the target DNA and the probe. Our data should help to optimise these methods. When designing the probe, a researcher should take into account that the binding site of Nt.BspD6I isoschizomers and neoschizomers (Nt.Bst9I, Nt.BstNBI, Nt.BhaIII, Nt.BstSEI, and Nt.MlyI) in DNA is probably ≈21 bp. For the most effective interaction of NE with DNA, at least 8 bp is required downstream of the strand cleavage site (on the 3′ side) in the duplex, and 4 bp is necessary on the 5′ side of the recognition site. As demonstrated here, the feasible detection of this interaction by means of a fluorescence change—due to a shortening of the distance between the fluorophore and quencher already at the stage of binding in the presence of Ca^2+^ ions—can also find practical applications in situations when the DNA cleavage is undesirable.

The main features of the proposed mechanism of Nt.BspD6I action are similar to the mechanism of type II REs (components of the restriction–modification systems that protect the bacterial cell from the entry of foreign DNA). This observation confirms the role of this enzyme as a component of the type II restriction–modification system of the bacterium *Bacillus* sp. D6 as suggested in ref. [7]. There is a structural similarity between Nt.BspD6I and R.FokI, which belongs to the IIS subtype of REs [8]. Nonetheless, the mechanism of Nt.BspD6I action has more in common with REs of the IIP subtype: EcoRI and EcoRV. Thus, just as R.EcoRI and R.EcoRV [64], Nt.BspD6I induces substrate bending during specific binding, while a DNA substrate in complex with R.FokI retains its canonical form [64]. Moreover, it has been shown elsewhere that Nt.BspD6I can serve as a large subunit of heterodimeric R.BspD6I and coordinate the small subunit of R.BspD6I (ss.BspD6I) on DNA; this process causes cleavage of the other substrate strand opposite to the nick introduced by Nt.BspD6I [10]. In other words, the interaction with R.BspD6I subunits can occur non-simultaneously for the top and bottom DNA strands. The formation of breaks in both strands of the DNA duplex promotes the dissociation of the enzyme–substrate complex [10]. R.FokI exists as a monomer in solution, just as Nt.BspD6I does, but it dimerises after DNA binding before the simultaneous cleavage of both its strands [30]. R.EcoRI and R.EcoRV usually function in the form of a homodimer [53], but for these enzymes, there is a possibility of accumulation of a DNA intermediate containing a single-strand break, which can then re-bind to the enzyme for the implementation of a double-strand break [26].

It is worth mentioning that the kinetic mechanism of Nt.BspD6I action is similar to the mechanism of AP endonucleases, which also introduce single-strand breaks into DNA. By contrast, they recognise an AP site (abasic site) or a nucleotide containing a damaged heterocyclic base in DNA. In this scenario, the enzyme also bends DNA, which takes place after initial damage-dependent DNA distortion [65]. For instance, the interaction of human AP endonuclease APE1 with a substrate also includes two-stage equilibrium binding, the irreversible formation of an enzyme–product complex, and equilibrium dissolution of this complex [66,67]. Thermodynamic analysis of these stages has made it possible to find out that the formation of a non-specific enzyme–substrate complex is accompanied by an increase in entropy and is driven either by the desolvation of polar groups in the protein–DNA contact area or by the displacement of highly ordered ‘crystalline’ water molecules from the DNA grooves [17]. Further research on the Nt.BspD6I interaction with a substrate is necessary to identify the differences and similarities in the mechanisms of action between this enzyme and AP endonucleases, which implement the same chemical reaction but differ in biological properties. 

Another class of NEs is MutL proteins from the mismatch repair system. The mechanism and specificity of their action in bacterial cells have not yet been unravelled [68]. Our findings can be useful in the search for answers to these questions, and in the future, they should help to create new sequence-specific NEs with various positions of phosphodiester bond hydrolysis in DNA.

## Figures and Tables

**Table 1:** DNA duplexes used in this work.

**DNA Duplex**	**Duplex Structure** **5** **′** **→3** **′** **3** **′** **→5** **′**	**Number of bp^a^ in Duplex**	**Number of bp after Cleavage Site**
**I-17**	GGTCTC**GAGTC**TTCT↓CA^b^ CCAGAG**CTCAG**AAGA-GT	17	2
**II-19**	GGTCTC**GAGTC**TTCT↓CAAG CCAGAG**CTCAG**AAGA-GTTC	19	4
**II-19A**	**FAM**-GGTCTC**GAGTC**TTCT↓CAAG^c^ CCAGAG**CTCAG**AAGA-GTTC	19	4
**II-19B**	GGTCTC**GAGTC**TTCT↓CAAG-BHQ1^d^ CCAGAG**CTCAG**AAGA-GTTC	19	4
**II-19C**	**FAM**-GGTCTC**GAGTC**TTCT↓CAAG-**BHQ1** CCAGAG**CTCAG**AAGA-GTTC	19	4
**II-19D**	GGTCTC**GAGTC**TTCT↓CAAG-**BHQ1** CCAGAG**CTCAG**AAGA-GTTC-**FAM**	19	4
**III-21**	GGTCTC**GAGTC**TTCT↓CAAGGT CCAGAG**CTCAG**AAGA-GTTCCA	21	6
**IV-23**	GGTCTC**GAGTC**TTCT↓CAAGGTAC CCAGAG**CTCAG**AAGA-GTTCCATG	23	8
**V-26**	CGTGGTCTC**GAGTC**TTCT↓CAAGGTAC GCACCAGAG**CTCAG**AAGA-GTTCCATG	26	8
**VI-30**	GCGTGGTCTC**GAGTC**TTCT↓CAAGGTACCTG CGCACCAGAG**CTCAG**AAGA-GTTCCATGGAC	30	11
**VI-30N**	GTATGAAGCTAGAGCCAGGTTGGCAGCATC CATACTTCGATCTCGGTCCAACCGTCGTAG	30	-

**Table 2:** Parameters of the Nt.BspD6I interaction with DNA duplex **VI-30** or **VI-30N**.

Constants	VI-30N, Ca^2+^	VI-30N, Mg^2+^	VI-30, EDTA
*k*_1_, μM^−1^ × s^−1^	63 ± 3	68 ± 6	83 ± 2
*k*_−1_, s^−1^	3.5 ± 0.7	4.6 ± 0.9	1.1 ± 0.2
*K_d_,* nM	60 ± 20	70 ± 20	13 ± 3

**Table 3:** Observed rate constants for the formation of Nt.BspD6I complexes with DNA duplexes of various lengths.

Length of DNA Duplex, bp	Number of bp Downstream of Cleavage Site	Mg^2+^		Ca^2+^	
		*k*_1_, s^−1^	*k*_2_, s^−1^	*k*_1_, s^−1^	*k*_2_, s^−1^
17	2	490 ± 6	128 ± 2	405 ± 6	8.1 ± 0.3
19	4	370 ± 4	28 ± 1	261 ± 4	6.2 ± 0.2
21	6	232 ± 4	21 ± 1	179 ± 4	15 ± 0.5
23	8	145 ± 2	10.0 ± 0.2	115 ± 2	7.7 ± 0.2
26	8	126 ± 1	7.0 ± 0.2	80 ± 1	6.4 ± 0.2
30	11	80 ± 1	10.0 ± 0.2	65 ± 1	7.0 ± 0.2

**Table 4:** Kinetic parameters of the interaction of some REs with short DNA substrates in steady-state settings.

Enzyme	Substrate Length, bp	Incubation Temperature, °C	*K_M_*, μM	*k_cat_*, s^−1^	Reference
Nt.BspD6I	19	37	0.10 ± 0.03	0.05 ± 0.01	present study
R.EcoRV	12	25	0.58 ± 0.06	0.70 ± 0.03	[27]
R.EcoRI	12	15	0.13 ± 0.09	0.011 ± 0.003	[61]
R.PaeR7	19	37	0.072 ± 0.008	0.30 ± 0.02	[62]

**Table 5:** Parameters of the interaction of Nt.BspD6I with DNA duplex **II-19C** or **II-19D**.

Constants *	II-19C, Ca^2+^	II-19C, Mg^2+^	II-19D, Mg^2+^
*k*_1_, μM^−1^ × s^−1^	120 ± 5	125 ± 7	24.3 ± 0.4
*k*_−1_, s^−1^	1.1 ± 0.8	20 ± 1	4.0 ± 0.1
*Kd* = *k*_−1_/*k*_1_, nM	9 ± 7	160 ± 20	165 ± 7
*k*_2_ (*k_cat_*), s^−1^		0.06 ± 0.01	0.06 ± 0.01
*k*_3_, s^−1^		0.62 ± 0.01	1.48 ± 0.01
*k*_−3_, μM^−1^ × s^−1^		0.041 ± 0.001	0.34 ± 0.01
*K*_M_ = (*k*_−1_+ *k*_2_)/*k*_1_, μM		0.16	0.17

* The parameters obtained in the presence of Ca^2+^ correspond to Scheme 1; the parameters obtained in the presence of Mg^2+^ match Scheme 2.

## Data Availability

Not applicable.

## Supplementary Materials

The following are available online at https://www.mdpi.com/article/10.3390/biom11101420/s1, Table S1: Wavelengths of fluorescence excitation and emission of the reporter groups, Table S2: Fluorescence change (rfu) corresponding to the Nt.BspD6I (1 μM) interaction with duplexes (1 μM) of different length in the presence of 10 mM CaCl2 and 10 mM MgCl2, Scheme S1, Figure S1: Nt.BspD6I structure (Protein Data Bank ID: 2EWF), Figure S2: Difference between one-step and two-step binding kinetic schemes for the Nt.BspD6I interaction with duplex VI-30 in the absence of divalent metal ions. Figure S3: Kinetic curves characterising the interaction of Nt.BspD6I (1 μM) with DNA duplexes (1 μM) of various lengths (indicated to the right of the curves) in the presence of 10 mM MgCl2. Figure S4: Analysis of the reaction mixtures (after hydrolysis of 120 nM DNA duplex II-19A with 7 nM Nt.BspD6I in the presence of 10 mM MgCl2) by electrophoresis in a 20% polyacrylamide gel with 7 M urea under denaturing conditions, Figure S5: Schemes of Nt.BspD6I interaction with DNA duplexes II-19C and II-19D, illustrating the effect of substrate conformation on fluorescence signal intensity (indicated by blue arrows) of the FAM/BHQ1 pair (fluorophore and fluorescence quencher), Figure S6: Kinetic curves describing the interaction of Nt.BspD6I (concentration is indicated to the right of the curves) with 1 µM duplex **II-19D** in the presence of 10 mM CaCl_2_.

## Abbreviations

BHQ1, Black Hole Quencher 1; bp, base pairs; dsDNA, double-stranded DNA; FAM, carboxyfluorescein; *K_d_*, dissociation constant; MMR, mismatch repair system; NE, N, nicking endonuclease (nickase); Nb, nicking endonuclease cleaving the bottom strand of double-stranded DNA; Nt, nicking endonuclease cleaving the top strand of double-stranded DNA; PAGE, polyacrylamide gel electrophoresis; RE, R, restriction endonuclease.
