# Supplementary material for: Kinetic Analysis of the Interaction of Nicking Endonuclease BspD6I with DNA
Source: Biomolecules. 2021 Sep 28;11(10):1420. doi: 10.3390/biom11101420 (PMC8533099; doi:10.3390/biom11101420)
Supplement: Supplementary file 1 [file biomolecules-11-01420-s001.zip › biomolecules-1343829-supplementary.pdf]

Supplementary Materials

Table S1. Wavelengths of fluorescence excitation and emission of the reporter groups.

Fluorophore or Quencher*	Excitation wavelength, nm	Emission wavelength, nm	Light filter
**Trp**	290	345	WG-320
**FAM**	494	525	OG-515
**BHQ1**	534	-	-

*The wavelength at the maximum of the optical excitation spectrum is given.

Table S2. Fluorescence change (rfu) corresponding to the Nt.BspD6I interaction with duplexes of different length in the presence of 10 mM $CaCl_2$ and 10 mM $MgCl_2$.

Length of DNA duplex, bp	Fluorescence change (rfu) in the presence of 10 mM $CaCl_2$	Fluorescence change (rfu) in the presence of 10 mM $MgCl_2$
17	0.076	0.088
19	0.075	0.111
21	0.099	0.122
23	0.105	0.118
26	0.097	0.112
30	0.156	0.174

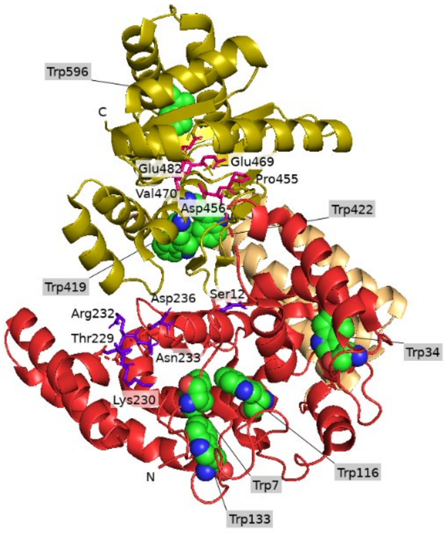

Figure S1. Nt.BspD6I structure (Protein Data Bank ID: 2EWF). DNA-binding domain is shown in red colour, linker domain – in peach colour, and catalytic domain – in olive colour. Trp residues are depicted in space-filling representation, the amino acids in a putative DNA-binding site are shown as violet sticks, and the amino acids in a putative catalytic site depicted as pink sticks. Proposed by us according to [Kachalova, G. et al. *J. Mol. Biol.* **2008**, *384*, 489–502].

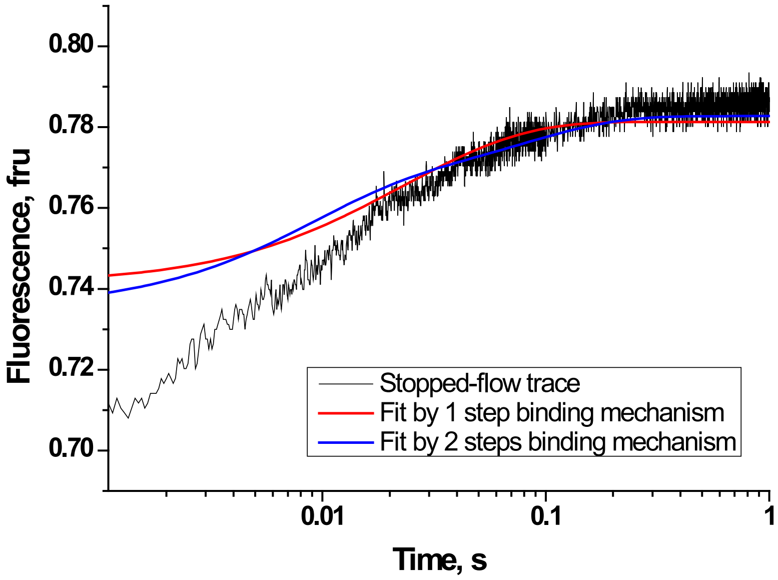

Figure S2. Difference between one-step and two-step binding kinetic schemes for the Nt.BspD6I interaction with duplex **VI-30** in the absence of divalent metal ions. The fit corresponds to [E] = 1.0 μM and [S] = 0.25 μM. For other substrate concentrations, there was no difference between the two kinetic schemes either.

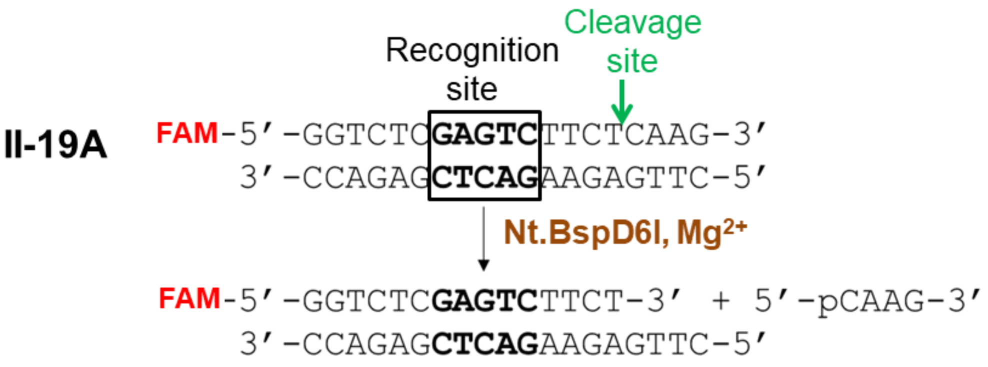

Scheme S1

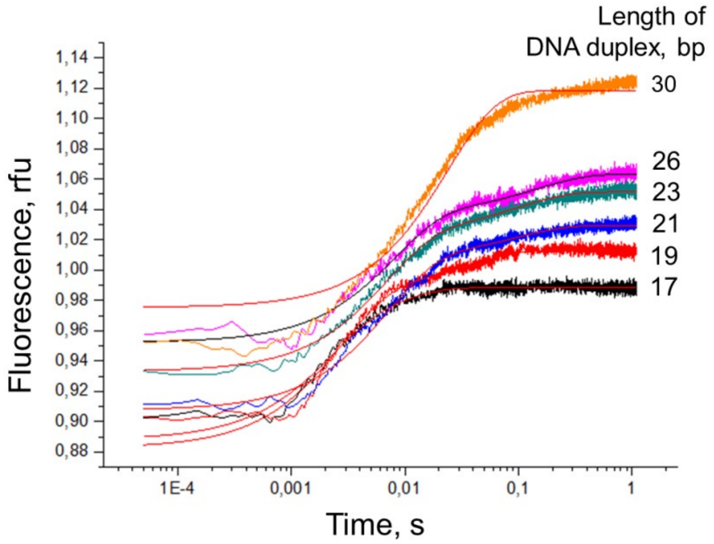

Figure S3. Kinetic curves characterising the interaction of Nt.BspD6I (1 μM) with DNA duplexes (1 μM) of various lengths (indicated to the right of the curves) in the presence of 10 mM $MgCl_2$. The coloured dots denote the experimental data, and each solid curve represents their fit to the equation $y = C + A_1e^{-k_1x} + A_2(1 - e^{-k_2x})$.

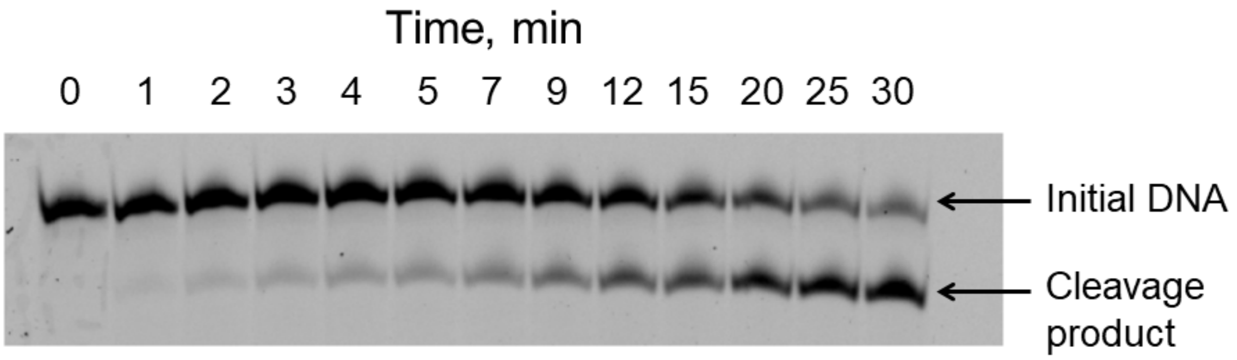

Figure S4. Analysis of the reaction mixtures (after hydrolysis of 120 nM DNA duplex **II-19A** with 7 nM Nt.BspD6I in the presence of 10 mM $MgCl_2$) by electrophoresis in a 20% polyacrylamide gel with 7 M urea under denaturing conditions. Bands containing DNA were detected via FAM fluorescence. Lane 0 contains only the original DNA.

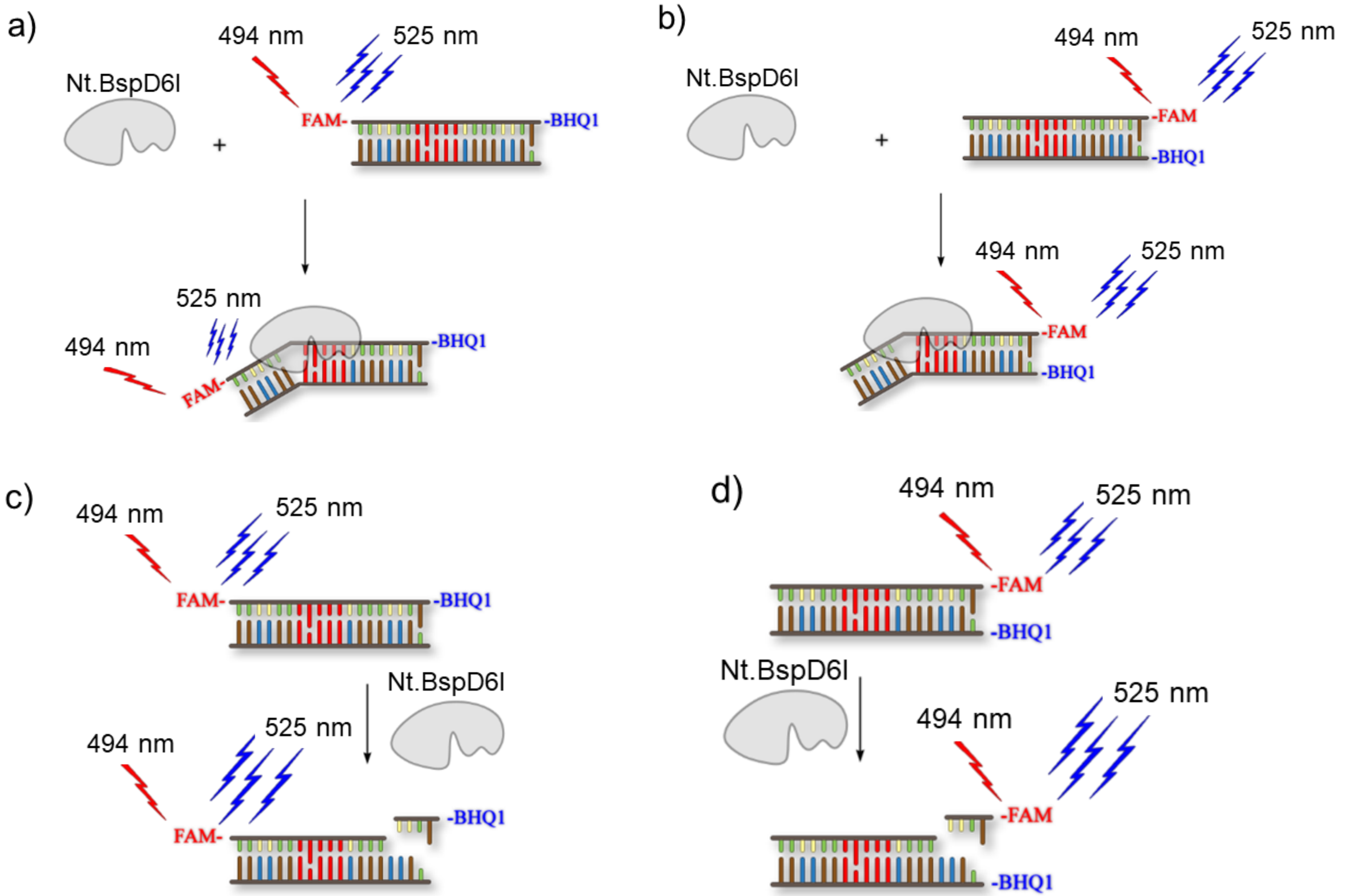

Figure S5. Schemes of Nt.BspD6I interaction with DNA duplexes **II-19C** and **II-19D**, illustrating the effect of substrate conformation on fluorescence signal intensity (indicated by blue arrows) of the FAM/BHQ1 pair (fluorophore and fluorescence quencher). Nt.BspD6I's recognition site is highlighted in red. (**a**) Bending of the **II-19C** substrate leads to a signal intensity decrease due to the approach of the fluorophore to the fluorescence quencher. (**b**) Nt.BspD6I binding with substrate **II-19D** will not induce a significant change in the fluorescence intensity. (**c**)–(**d**) The dissociation of the product of cleavage of DNA duplex **II-19C** or **II-19D** from the complex with NE will enhance the fluorescence intensity

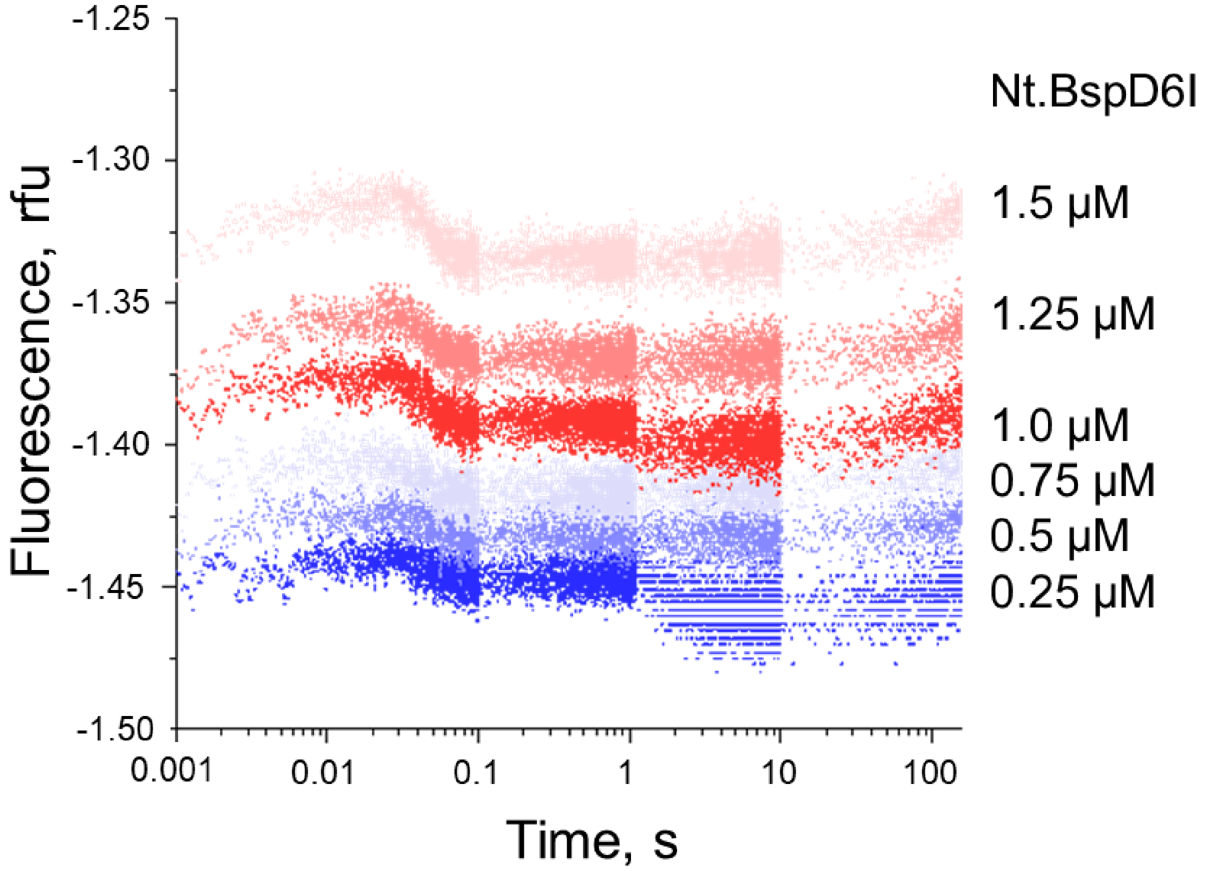

Figure S6. Kinetic curves describing the interaction of Nt.BspD6I (concentration is indicated to the right of the curves) with 1 μM duplex **II-19D** in the presence of 10 mM $CaCl_2$. Rfu: relative fluorescence units.
